# Evaluation of a menstrual hygiene intervention in urban and rural schools in Bangladesh: a pilot study

**DOI:** 10.1186/s12889-022-13478-1

**Published:** 2022-06-02

**Authors:** Mahbub-Ul Alam, Farhana Sultana, Erin C. Hunter, Peter J. Winch, Leanne Unicomb, Supta Sarker, Mehjabin Tishan Mahfuz, Abdullah Al-Masud, Mahbubur Rahman, Stephen P. Luby

**Affiliations:** 1 Environmental Interventions Unit, Infectious Disease Division, icddr,b, 68, Shaheed Tajuddin Ahmed Sarani, Mohakhali, Dhaka, Bangladesh; 2 School of Public Health, Faculty of Medicine and Health, The University of Sydney, Sydney, NSW Australia; 3 Department of International Health, Johns Hopkins Bloomberg School of Public Health, Baltimore, USA; 4 Division of Infectious Diseases & Geographic Medicine, Stanford University, Stanford, USA

**Keywords:** Menstrual hygiene management (MHM), Adolescence, Bangladesh, Menstrual hygiene intervention, School absenteeism, Menstrual health and hygiene

## Abstract

Girls’ menstrual experiences impact their social and educational participation, physical and psychological health. We conducted a pilot study to assess the acceptability and feasibility of a multi-component intervention intended to support menstruating girls; improve menstrual care knowledge, practices, and comfort; and increase school attendance.

We conducted a pre/post evaluation of a 6-month pilot intervention in four schools (2 urban, 2 rural) in Dhaka, Bangladesh. We selected 527 schoolgirls (grades 5 to 10; aged 10 to 17 years) for a baseline survey and 528 girls at endline. The intervention included: 1) Menstrual Hygiene Management (MHM) packs– reusable cloth pads, underwear, carry bags and menstrual cycle tracking calendars, 2) education curriculum- pictorial flipcharts, puberty related-booklets, and teachers’ training to deliver puberty and MHM sessions, 3) maintenance- improvements to school sanitation, provision of disposable pads in the school office, provision of chute disposal systems for disposable pads, and gender committees to promote a gender-friendly school environment and maintenance of intervention facilities. We estimated intervention uptake and intervention effect by calculating prevalence differences and 95% confidence intervals using fixed-effects logistic regression.

The intervention uptake was more than 85% for most indicators; 100% reported receiving puberty education, 85% received MHM packs, and 92% received booklets. Reusable cloth pads uptake was 34% by endline compared with 0% at baseline. Knowledge about menstrual physiology and knowledge of recommended menstrual management practices significantly improved from baseline to endline. Reported improvements included more frequent changing of menstrual materials (4.2 times/day at endline vs. 3.4 times/day at baseline), increased use of recommended disposal methods (prevalence difference (PD): 8%; 95% Confidence Interval: 1, 14), and fewer staining incidents (PD: − 12%; 95% CI: − 22, − 1). More girls reported being satisfied with their menstrual materials (59% at endline vs. 46% at baseline, *p* < 0.005) and thought school facilities were adequate for menstrual management at endline compared to baseline (54% vs. 8%, *p* < 0.001). At endline, 64% girls disagreed/strongly disagreed that they felt anxious at school due to menstruation, compared to 33% at baseline (*p* < 0.001). Sixty-five percent girls disagreed/strongly disagreed about feeling distracted or trouble concentrating in class at endline, compared to 41% at baseline (*p* < 0.001). Self-reported absences decreased slightly (PD: − 8%; 95% CI: − 14, − 2).

Uptake of cloth pads, improved maintenance and disposal of menstrual materials, and reduced anxiety at school suggest acceptability and feasibility of the intervention aiming to create a supportive school environment.

**Supplementary Information:**

The online version contains supplementary material available at 10.1186/s12889-022-13478-1.

## Introduction

Research in low- and middle-income countries (LMICs) has highlighted how girls’ menstrual experiences impact their social participation, participation in education, and their psychological and physical health [1]. Girls’ menstrual experiences are influenced by their physical and economic environments, as well as their access to knowledge about menstruation and social support [1]. Schoolgirls who menstruate require access to clean and reliable materials to absorb or collect menses and private and safe sanitation facilities to enable changing and washing or disposal of used menstrual materials and washing of the body with soap and water [2]. Timely and accurate information about menstruation and its management as well as supportive social environments free from menstrual stigma could improve the ability of girls to manage menstruation [3]. Ensuring that students are able to address their menstrual hygiene needs in the school environment is integral to achieving multiple sustainable development goals—including good health and wellbeing, quality education, and gender equality, among others [4]. However, global evidence suggests that schools in resource-constrained settings are rarely supportive of these requirements [5, 6].

In 2014, UNICEF and Columbia University organized the “MHM in Ten” working group to establish a 10-year agenda for achieving the vision that by 2024 “girls around the world are knowledgeable about and comfortable with their menstruation, and are able to manage their menses in school in a comfortable, safe, and dignified way” [7]. The working group’s first priority for achieving the vision was to establish a strong cross-sectoral evidence base for menstrual hygiene management (MHM) in schools so that effective policies, resource allocation, and programming at scale may be prioritized appropriately [7]. Advocacy efforts began increasingly drawing attention to the need to support menstruating students. Yet, a 2016 systematic review and meta-synthesis concluded that there was insufficient evidence to establish the effectiveness of menstrual management interventions in improving women and girls’ education, work, and psychosocial wellbeing in LMIC [8]. Eight intervention trials were identified, but the review authors noted a high risk of bias across studies. Although the included studies suggested some benefits of these programs, more rigorous evidence is required to better inform policy and program development and resource allocation moving forward [8].

In Bangladesh, the first acknowledgment and endorsement of the need for good menstrual hygiene management practices for schoolgirls by the Government of Bangladesh (GoB) came in a 2015 circular distributed to all district and sub-district level education officers. The circular called for the improvement of school toilets, including the creation of separate toilet facilities for female students, the provision of soap, water and waste bins and the appointment of teachers to serve as focal points for educating schoolgirls about menstruation [9]. This decision was informed by the 2014 Bangladesh National Hygiene Baseline which found that only 6% of schools nationwide provided education about menstrual hygiene, 41% of girls reported missing school during their menstrual periods in the preceding 3 months (average 3 days missed per period), and 32% reported that menstruation negatively affected their school performance [5]. However, implementation of the circular’s recommendations has been limited. The 2018 National Hygiene Survey found that 30% of adolescent schoolgirls still report missing school during their menstrual periods, for an average of 2.5 days missed per period [10].

To respond to the GoB circular’s recommendations and contribute to progress towards the “MHM in Ten” vision, we developed and piloted a multi-component intervention intended to improve schoolgirls’ menstrual experiences and ensure school environments are supportive. We aimed to assess the acceptability and feasibility of the pilot intervention to inform a future cluster-randomized control trial that would enable us to rigorously assess the intervention package’s effects on schoolgirls’ psychosocial wellbeing and education outcomes.

Our formative research (manuscript under development) in urban and rural schools in Bangladesh showed that social proscriptions on discussing puberty and menstruation contributed to students’ limited knowledge about the physiological basis for their menstrual cycle. Girls complained about unsupportive school environments that lacked clean and sufficient toilet facilities, water, soap, privacy, and waste disposal options, which made it difficult or impossible for some to change their menstrual materials at school. Consequently, many girls reported waiting until they returned home to change their soaked menstrual materials or staying home from school during the heaviest days of bleeding unless they had important exams or review sessions. Students also discussed the stigma and shame associated with menstruation, the fear of ridicule from teachers and other students if menstrual blood stained their uniform or school benches, and the distraction this caused from their studies.

Guided by our formative research findings and an intervention development workshop convening stakeholders from government, NGOs, development partners, academic partners, and icddr,b, we developed and pre-tested individual intervention components (presented separately) before finalizing the multi-component intervention for piloting. The objectives of the pilot study were to: 1) assess the acceptability and feasibility of implementing the intervention package among both girls and boys, which focused on both menstrual practices and puberty education, 2) develop and test new strategies for assessing the impact of school-based interventions on school attendance, academic performance, and self-efficacy in addressing menstrual needs, and 3) evaluate intervention outcomes at baseline and endline (knowledge about menstrual physiology; knowledge of recommended menstrual management practices; girls’ perceptions of their environment, practices, and comfort; reported menstrual management practices; and school absenteeism). Results from the study’s evaluation of new strategies for assessing school attendance, academic performance, and self-efficacy are presented elsewhere (manuscripts under development). Here, we present the quantitative assessment of the pilot intervention’s: a) acceptability and feasibility, and b) changes in menstruation-related knowledge, practices, perceptions, and self-reported school absenteeism from baseline to endline among girls as indicators of potential for the intervention’s impact.

## Methods

### Study design and sample selection

We conducted a pre (01 to 15 August 2017) and post-test (10–25 April 2018) evaluation of a pilot intervention package in four schools in Dhaka Division, Bangladesh (2 urban, 2 rural). Our study team collected a list of schools in urban Dhaka from the Divisional Education Department and in rural Manikganj District from the office of sub-district level Administrative Education Officers. Field Research Assistants telephoned 200 schools and identified 40 that met our inclusion criteria (Table 1). We visited 20 randomly selected schools out of the 40, and from those purposively selected 8 schools (4 for formative research phase, 4 for intervention piloting) to include both public and private schools in urban and rural settings. The Dhaka Zonal Office, Directorate of Secondary and Higher Education; the Dhaka Divisional Office, Directorate of Primary Education; and School Management Committees (SMC) provided permission for the research to take place in the participating schools. The intervention was designed following an iterative approach which will be described in our formative paper (in progress). The findings of our formative research were consistent with results of the 2014 and 2018 Bangladesh NHS. The formative research indicated lack of MHM products, disposal and WASH facilities in schools and revealed that the schoolgirls preferred reusable cloth pads (Sultana, unpublished data), chute disposal system [11], and demonstrated that a fingerprint device would be an accurate system to measure absenteeism data (Sultana, unpublished data).

**Table 1:** Inclusion criteria for school selection

1. Co-educational (girls and boys studying in the same school)	
2. Offer grades 1–10 (approx. Ages 6–17 years)	
3. No ongoing water, sanitation, or menstrual hygiene program or intervention by other organisations	
4. Presence of functional toilet(s) on-site	
5. At least one male and one female teacher	

### Sample size calculation

Based on the objectives, we used multiple indicators (e.g., menstruation knowledge, practices, attendance, and school achievement) to calculate the sample size. Based on clustering in schools, we determined sample size using proportions of these indicators from the 2014 Bangladesh National Baseline Survey. We assumed a minimum detectable difference of 14% for all indicators and estimated that we needed 509 respondents, using a 0.03 Intra-class correlation (ICC), 2.47 design effect [5] and 10% non-response.

### Intervention delivery

The study team convened meetings with teachers, School Management Committee and Parent Teacher Association members at each participating school to provide overviews of the intervention and address questions before commencement of the pilot intervention. The team held parent conferences in the beginning of the study to make the parents aware about the components, the interventions, and the objective of the study. The intervention (Supplementary Table 1) was implemented by the schools with the support of the icddr,b study team, which comprised members with expertise in public health, medicine, health promotion and education, anthropology, and psychology.

#### Puberty education

**Fig. 1:**
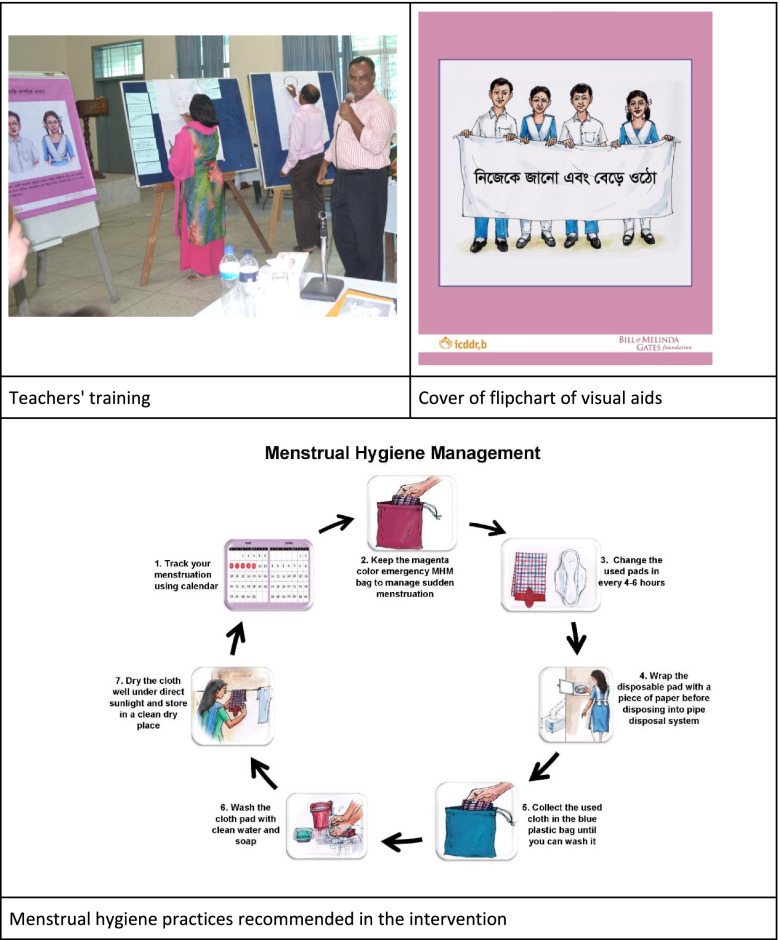
Education curriculum and visual aids

We asked the headteacher at each school to nominate at least one male and one female teacher from grades 5–10 to implement an interactive puberty education curriculum for their students. The curriculum entitled *Know Yourself & Grow* comprised four modules: 1) growing up, 2) reproductive systems, 3) menstruation, and 4) nutrition. We applied a training of trainers model to provide 18 schoolteachers (including headteachers) a 3-day residential training on how to deliver the *Know Yourself & Grow* curriculum and another 2-day mid-intervention refresher training. Participating teachers were drawn from teachers of physical education, science, or home economics. At the conclusion of the training of trainers, teachers provided individualized delivery plans for completing all modules in their schools over the 6-month pilot period. Teachers delivered the content of the four modules to their students in varying numbers of sessions according to what was feasible in their context. We provided each teacher with a teacher’s manual, locally illustrated flip charts to use as visual aids while conducting education sessions (Fig. 1), and an electronic slide deck of the materials.

The content of the puberty education was obtained from the government approved textbooks and then expanded to include more detailed content drawn from puberty booklets that had been previously developed by Bangladesh Center for Communication Programs (BCCP). We added additional content and activities regarding the practical aspects of menstrual hygiene management, pain mitigation, and addressing menstrual stigma which was not in the existing textbooks. The intervention was developed with consent and guidance from the Directorate of Secondary and Higher Education (DSHE) and MHM Working Group. The curriculum relied heavily on participatory activities, games, and role-plays to increase comfort in discussing menstruation and reduce stigma. This provided opportunities for behaviour modelling and practicing new skills (e.g., asking for support from school staff, affixing menstrual materials to underwear, estimating next menstrual period based on menstrual cycle length, proactively supporting their peers, managing menstrual pain etc.). After the training of trainers, we also provided a 1-day orientation on the schools’ premises to orient all other teachers to the material that their students would be receiving from the intervention.

To supplement the in-class education sessions, we provided each student a set of three booklets previously developed by the Bangladesh Center for Communication Programs: one with information about adolescence and puberty, one about a boy experiencing nocturnal emission for the first time and a storybook about a girl experiencing her first menstrual period [12].

#### Question box

Classrooms were outfitted with a locked “question box” for students to submit questions that they felt uneasy to ask in class. Teachers were to provide answers in subsequent sessions according to the information provided in their teacher’s manual. Study team members collected all submitted questions at the end of the pilot period for documentation and provided a curated list of responses to common questions for the teachers to use as a resource in the future.

#### MHM packs

**Fig. 2:**
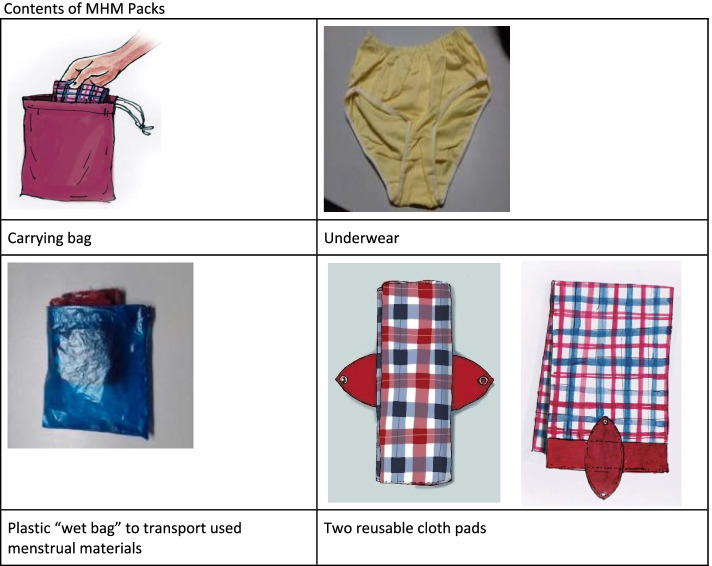
Contents of MHM Packs

With support from school staff, study team members distributed MHM packs to every girl in grades 6–10 and to girls in grade 5 who had reached menarche. We left extra materials in school offices for students who were absent during distribution days. MHM packs (Fig. 2) comprised a carrying bag containing two reusable cloth pads (Sultana icddr,b Reusable Cloth Pad, designed by F.S. and the team, and pre-tested during the study’s formative research phase), one plastic “wet bag” to carry used menstrual materials, one underwear, and a menstrual tracking calendar (Fig. 2). We also supplied schools with a stock of disposable menstrual pads (provided by a local company named Social Marketing Company) [13] for girls who began menstruating suddenly at school and needed quick access to absorbents. The disposable pads were counted and handed to the janitors responsible for maintaining the adolescent corner of each school. We also provided them with a registrar book. If a student needed to access disposable pads, the student asked the responsible janitor who noted the student’s name, the number of pads taken, the date along with a signature from the receiving student. Study team members provided hands-on practical training to students on how to use the materials in the MHM packs at the time of distribution, and further participatory training and practice was built into the puberty education sessions facilitated by schoolteachers.

#### Improvements to sanitation facilities

We made minor improvements to schools’ sanitation facilities according to requirements by school students, such as fixing toilet cubicle doors, locks, and lights. We installed a chute disposal system for used menstrual materials in one female toilet cubicle in each school [11]. The chute disposal system was modelled after a design created by WaterAid in Bangladesh [14] and chosen by the students as a preferred method of disposal during the pre-testing phase of the study. We encouraged schools to provide pieces of scrap newspaper in the toilets for students to wrap used menstrual pads before disposal and soap for handwashing. We affixed posters in the girls’ toilets that illustrated the proper use of the disposal system and recommended MHM practices (Fig. 1) [11].

#### School gender committees

Each school formed a gender committee consisting of a female and a male student representative from each grade, teachers, headteacher, janitor, a member of the School Management Committee and the Upazila Nirbahi Officer (an administrative authority for the sub-district). Gender committees met monthly to discuss issues concerning the distributed menstrual materials, education sessions, school toilet conditions and required improvements.

### Evaluation methods

#### Pre/post survey

We formed a separate team for survey data collection and arranged training for them. Before implementation of the pilot intervention, we selected 527 girls randomly from the participating schools’ rosters (out of those present on the day) in grades 5 to 10 to participate in a baseline survey. After 6 months of piloting the intervention package in the 4 schools, 528 randomly selected girls, selected similarly from schools’ rosters from grades 5 to 10 participated in the endline survey. We tried to select equal number of students from each grade from the rosters. We administered a tablet-based structured questionnaire for data collection.

#### Outcome measures

We assessed the intervention package’s acceptability and feasibility by reporting intervention uptake measures, particularly whether girls received and used intervention materials. Secondary outcomes included changes in knowledge about menstruation and recommended menstrual management behaviours; menstrual care practices; girls’ perceptions of their environment, practices, and comfort; and self-reported school absenteeism during most recent menstrual period.

We assessed knowledge about menstrual physiology on five indicators and we reported how many girls responded to them correctly. Knowledge of recommended menstrual management practices was based on four indicators, and we reported how many girls responded to them correctly. Girls’ perceptions of their environment, practices, and comfort were captured by using Likert items.

#### Data management and analysis

We reported prevalence difference (PD) to measure the intervention uptake and other outcomes. Intervention effect on binary outcomes was estimated by comparing endline versus baseline measures, using prevalence differences (PD) and 95% confidence intervals (CI) estimated using fixed-effects logistic regression. Effects on continuous outcomes were similarly assessed with fixed-effects linear regression to estimate the mean difference and 95% confidence intervals (CI). We adjusted for clustering (schools) effect using Sandwich estimator. For data captured using Likert items, we calculated a Chi-square test for linear trend (Cochran–Armitage test for linear trend) [15] to examine changes in these ordinal variables from baseline to endline.

### Stakeholder engagement

We formed an “MHM working group” in June 2017 involving stakeholders from the Ministries of Education, and Health and Family Welfare; Directorate General of Health Services; Directorate of Primary, Madrassa, Secondary and Higher, and Technical Education; Education Engineering Department; Department of Public Health Engineering; Bangabandhu Sheikh Mujib Medical University (BSMMU); Shornokishoree Network Foundation; and NGOs working on MHM issues. The working group intended to involve relevant stakeholders in the development of the pilot intervention and to motivate educators and policymakers in Bangladesh to implement a nationwide menstrual health and hygiene strategy. We held quarterly meetings to inform members of study updates and discussed ways forward.

### Ethical approval

The study protocol was reviewed and approved by the Ethical Review Committee of icddr,b in Dhaka, Bangladesh. We sought written approval from Directorate of Primary Education, Directorate of Secondary and Higher Education, and School Management Committees (SMC) of the participating schools for the research to take place. The study team convened meetings with Headmaster, teachers, School Management Committee and Parent Teacher Association members at each participating school to provide overviews of the study and addressed questions before commencement of the pilot intervention. We obtained written informed assent from the students and the schoolteachers provided written informed consent for the student’s participation as their guardians (in loco parentis). Each students were informed about the purpose of the study, data collection procedures and time requires for the survey, risks and benefits of participating in this study, privacy, anonymity and confidentiality of the data before beginning the questionnaire. Additionally, they were reminded that they could withdraw their participation at any time or refuse to answer any question they did not want to answer.

## Results

### School characteristics

One of the four intervention schools was a government school, and the other three were non-governmental. Although all schools were co-educational, the two urban schools scheduled different shifts for boys and girls. The participating schools served between 940 and 2100 students. The two rural schools had twice the number of male teachers as females, while urban schools had nearly equal or slightly more female teachers than male. The ratio of toilets designated for use by girls to the number of female students in each school ranged from 1:27 to 1:250. Only one of the four schools at baseline provided waste disposal bins in student toilets.

### Participant characteristics

There was no refusal of assent during the baseline or endline survey. Overall, the mean age of girls and the household characteristics were similar at baseline and endline (Table 2).

**Table 2:** Characteristics of adolescent schoolgirls from four rural and urban schools of Bangladesh at baseline and endline

Indicators	Baseline (***N*** = 527) n (%)	Endline (***N*** = 528) n (%)
Age of respondent in years (mean, SD)	13.8 (1.9)	13.4 (1.9)
Respondent’s education:		
Mean grade level (mean, SD)	7.6 (1.7)	7.6 (1.7)
Education of mother of the respondent:		
No education	60 (11)	43 (8)
Grade 1–5	122 (23)	108 (21)
Grade 6–10	203 (39)	214 (41)
Above grade 10	56 (11)	76 (14)
Do not know	86 (16)	87 (17)
Education of father of the respondent:		
No education	67 (13)	55 (10)
Grade 1–5	58 (11)	45 (9)
Grade 6–10	160 (30)	150 (28)
Above grade 10	99 (19)	119 (23)
Do not know	143 (27)	159 (30)
Main occupation of father of the respondent:		
Business/shopkeeper/ambulant vendor	176 (33)	191 (36)
Salaried job	127 (24)	152 (29)
Farmer/Cultivator	56 (11)	53 (10)
Skilled worker	55 (10)	51 (10)
Van/Rickshaw puller	39 (7)	22 (4)
Staying abroad	31 (6)	36 (7)
Died/Untraced/ unemployed/disabled	31 (6)	19 (4)
Others (politician, homeopathic doctors and don’t know)	12 (2)	4 (1)
Main occupation of mother of the respondent:		
Homemaker/housewife	450 (85)	456 (86)
Salaried job	24 (6)	51 (10)
Shopkeeper/Business/ambulant vendor/tailor	14 (3)	10 (2)
Domestic maid/labour	11 (2)	5 (1)
Staying abroad	4 (1)	3 (1)
Died/untraced	10 (2)	3 (1)

### Intervention delivery and uptake

Menstruation education sessions were provided in the schools and were reported by 100% of the students. Ninety-two percent of schoolgirls reported receiving puberty information booklets and among them, 96% reported having read them (Table 3).

**Table 3:** Intervention delivery and uptake of the intervention at endline in four urban and rural schools of Bangladesh as reported by female students, 2018

Indicators	Urban		Rural		Endline ***N*** = 528 n (%)
	Tejgaon govt. high school (***N*** = 132) n (%)	National bangla high school (***N*** = 132) n (%)	Oxford high school (***N*** = 132) n (%)	Kathigram high school (***N*** = 132) n (%)	
MHM taught in school:	132 (100)	132 (100)	132 (100)	132 (100)	528 (100)
Knew about gender committee	70 (53)	98 (74)	96 (73)	98 (74)	362 (69)
Heard about chute disposal system for menstrual materials	75 (76)	91 (90)	86 (97)	87 (94)	339 (64)
Used chute disposal system	18 (24)	23 (25)	37 (43)	33 (38)	111 (33)
Reason for not having used chute disposal system (multiple responses)	*N* = 75	*N* = 91	*N* = 86	*N* = 87	*N* = 339
Did not know about chute disposal system	57 (76)	68 (75)	49 (57)	54 (62)	189 (56)
Did not change menstrual materials at school	51 (68)	63 (69)	45 (52)	49 (56)	208 (61)
Used reusable menstrual materials	5 (6.7)	11 (12)	7 (8.1)	8 (9.2)	31 (9.1)
Problem with chute disposal system	5 (6.7)	2 (2.2)	1 (1.2)	2 (2.3)	10 (2.9)
Did not know how to use chute disposal system/disposed elsewhere	1 (1.3)	1 (1.1)	0 (0)	1 (1.1)	4 (1.2)
Knew about question box	128 (97)	127 (96)	128 (97)	122 (92)	505 (96)
Submitted question into question box	15 (12)	41 (32)	34 (27)	19 (16)	109 (21)
Received answer to submitted question	10 (67)	31 (76)	27 (79)	9 (47)	77 (71)
Received puberty information booklet	124 (94)	125 (95)	108 (82)	131 (99)	488 (92)
Read puberty information booklet	118 (95)	123 (98)	102 (94)	126 (96)	469 (96)
**Materials for menstruation, regarding menstrual materials distributed to menstruating girls:**	*N* = 99	*N* = 101	*N* = 89	*N* = 93	(*N* = 382)
Received carry bag for menstrual materials	73 (74)	94 (93)	74 (83)	82 (88)	323 (85)
Used carry bag	55 (75)	67 (71)	63 (85)	57 (69)	242 (75)
Received plastic “wet bag” for transporting used menstrual materials	77 (78)	91 (90)	73 (82)	81 (87)	322 (84)
Used the wet bag to transport used menstrual materials	44 (57)	39 (43)	54 (74)	33 (41)	170 (52)
Obtained disposable menstrual pad for emergency use at school	45 (45)	32 (32)	10 (11)	13 (14)	100 (26)
Used disposable menstrual pad obtained from school office	39 (87)	30 (94)	10 (11)	13 (14)	92 (92)
Average number of disposable pads obtained by the girl (mean, SD) (*n* = 100)	1.6 (0.5)	1.3 (0.7)	1.8 (1.1)	1.3 (0.6)	1.5 (0.7)
Received underwear	*N* = 99 76 (77)	*N* = 101 95 (94)	*N* = 74 74 (83)	*N* = 93 82 (88)	*N* = 367 327 (86)
Used underwear	58 (76)	75 (79)	72 (97)	61 (74)	266 (81)
Received reusable cloth pad	78 (79)	95 (94)	74 (83)	84 (90)	331 (87)
Used reusable cloth pad	52 (67)	73 (77)	64 (86)	63 (75)	252 (76)
Received menstrual tracking calendar	*N* = 99 81 (82)	*N* = 102 94 (92)	*N* = 88 78 (89)	*N* = 93 84 (90)	*N* = 382 337 (88)
Used menstrual tracking calendar	65 (80)	72 (77)	66 (85)	59 (70)	262 (78)

Ninety-six percent of students reported being aware of question boxes and 21% of the students had submitted questions in the boxes, among which 71% received answers to their questions. Among 382 post-menarcheal girls, 85% received a carrying bag for menstrual materials, 75% of which used them. Eighty-four percent of girls received plastic “wet bags” for transporting used menstrual materials, and 52% of those used them. Twenty-six percent of girls obtained disposable menstrual pads for emergency use at school. Eighty-six percent of girls received underwear and 81% of those used them. Eighty-eight percent of girls received a menstrual tracking calendar, and 78% of those used the calendar to track their menstrual cycle (Table 3).

### Knowledge about menstruation and recommended menstrual management practices

More schoolgirls correctly responded to four of the five menstrual physiology knowledge items during endline (10%) compared to baseline (2%) (PD: 8; 95% CI: 5, 11). More schoolgirls were able to correctly respond to three out of the four questions about recommended menstrual management during endline (80%) compared to baseline (35%) (PD: 45; 95% CI: 40, 51). More schoolgirls knew at least three methods for reducing pain or physical discomfort during menstruation at the endline (28%) compared to baseline (10%) (PD: 18; 95% CI: 14, 23). (Table 4).

**Table 4:** Adolescent girls’ knowledge about menstrual physiology and knowledge of recommended menstrual management practices before and after the intervention in four urban and rural schools of Bangladesh, 2018 (Reported)

Indicators	Baseline n (%)	Endline n (%)	Prevalence difference (PD) and 95%CI
**Knowledge about menstrual physiology:**			
Mentioned 12 years as the average age of menarche in Bangladesh	217 (41)	199 (38)	-3 (−7, 0)
Mentioned “to shed the lining of the uterus” as reason for the menstrual period	95 (18)	190 (36)	18 (9, 26)
28 days as average length of the menstrual cycle	113 (21)	287 (54)	31 (15, 48)
3–7 days as typical duration of each period of bleeding	379 (72)	343 (65)	−7 (−14, 0)
Girls could identify all parts of the female reproductive system^a^	4 (1)	70 (13)	18 (6, 31)
Girls responded with 4 out of 5 above responses as correct (Excluding Girls could identify all parts of the female reproductive system)	10 (2)	53 (10)	8 (5, 11)
**Knowledge about recommended menstrual management practices:**			
Knew at least 3 methods^b^ for reducing pain or physical discomfort during menstruation	52 (10)	149 (28)	19 (13, 24)
“Wash with water and soap and dried under sunlight” as recommended method to wash and dry reusable menstrual materials	276 (52)	463 (88)	33 (29, 38)
“Dispose in the waste bin/burned/bury or used chute disposal system” as appropriate disposal methods for disposing of single-use menstrual materials	410 (78)	519 (98)	27 (7, 46)
“At least every 6 hours” as appropriate frequency of changing menstrual materials	333 (63)	447 (85)	21 (10, 32)
Girls who correctly responded to 3 out of the 4 items above	183 (35)	422 (80)	45 (40, 51)

^a^Ovaries, fallopian tubes, uterus, and vagina

^b^Recommended methods were pain medication, exercise, hot fomentation

### MHM practices

Although disposable pads were the most used menstrual materials, reusable cloth pads were taken up by 34% of the girls by endline compared with 0% at baseline (Table 5). At endline, the most reported reasons for using the reusable cloth pads were because they were “easy to use” (41%) and “washable” (32%).

**Table 5:** Menstrual hygiene practices among adolescent girls before and after intervention from four urban and rural schools of Bangladesh, 2018 (Reported)

Indicators	Baseline n (%)	Endline n (%)	Prevalence difference (CI)
Absorbent used during last menstrual period while inside the home (multiple responses):	*N* = 404	*N* = 382	
disposable pad	276 (68)	236 (62)	−7 (− 15,2)
scrap cloth	170 (42)	93 (24)	−17 (−24, − 11)
tissue/cotton wool/fabric scraps from garment factory	25 (6)	29 (8)	1 (−2, 5)
reusable cloth pad	0	129 (34)	34 (29, 38)
Absorbent used during last menstrual period while inside the home (Single response**)**			
disposable pad only	211 (52)	161 (42)	−10 (−16, −4)
scrap cloth only	117 (29)	51 (13)	−16 (−23, −8)
tissue only	5 (1)	2 (1)	−1 (−2, 1)
cotton wool only	0	1 (0.3)	0 (0, 1)
fabric scraps from garment factory only	2 (0.5)	1 (0.2)	0 (−1, 0)
reusable cloth pad only	0	67 (18)	18 (14, 21)
scrap cloth and reusable cloth pad	0	16 (4)	6 (3, 8)
scrap cloth and disposable pad	50 (13)	19 (5)	−9 (−15, 0)
disposable pad and reusable cloth pad	0	35 (9)	9 (6, 12)
disposable pad and tissue	13 (3)	13 (3)	0 (−3, 3)
Absorbent used during last menstrual period while outside the home (multiple responses):			
disposable pad	320 (79)	269 (70)	−9 (−13, −4)
scrap cloth	88 (22)	58 (15)	−7 (−8, −5)
tissue/cotton wool/fabric scraps from garment factory	27 (7)	22 (6)	−1 (− 5, 3)
reusable cloth pad	0	99 (26)	26 (22, 30)
Absorbent used during last menstrual period while outside the home (single responses):			
disposable pad only	279 (69)	219 (57)	−12 (−15,−8)
scrap cloth only	63 (16)	31 (8)	-8 (−13, −2)
tissue only	2 (1)	2 (1)	0 (−1, 1)
reusable cloth pad only	0	54 (14)	14 (11, 18)
cotton only	0	1 (0.3)	0 (0, 1)
fabric scraps from garment factory only	2 (1)	1 (0.3)	0 (−1, 0)
scrap cloth and reusable cloth pad	0	14 (4)	4 (2, 6)
scrap cloth and disposable pad	21 (5)	11 (3)	−2 (−7, 3)
scrap cloth and cotton	0	0	−1 (−1, 1)
disposable pad and reusable cloth pad	0	13 (3)	3 (1, 5)
disposable pad and tissue	11 (3)	11 (3)	0 (−3,4)
Reasons to use reusable cloth pads:	*N* = 72	*N* = 246	
Easy to use	17 (24)	100 (41)	18 (13, 24)
Washable	24 (33)	78 (32)	−2 (−11, 7)
Reduce germs/urinary tract infections	10 (14)	20 (8)	−5 (−10,0)
Easy to put on and off/dispose	6 (8)	19 (8)	−1 (−5,4)
Low cost and available	5 (7)	8 (3)	−3 (−7, 1)
More privacy	4 (6)	10 (4)	−1 (−6, 3)
No need to wash	3 (4)	0	−4 (−2, −7)
Number of times per day girls typically changed menstrual materials during last menstrual period (mean, SD)	3.4 (1.3)	4.2 (1.6)	0.79 (0.52,1.05)
Longest time (hours) spent without changing menstrual material during last menstrual period	11 (3.5)	8 (3.3)	−3.2 (−3.9, −2.5)
Disposed the disposable pad during last menstrual period: (multiple responses allowed)	*N* = 319	*N* = 276	
In waste bin/chute disposal system	174 (55)	172 (62)	8 (1, 14)
Other than waste bin^†^	76 (24)	50 (18)	−6 (−13,2)
Burried at home	74 (23)	62 (23)	−1 (−4, 3)
Washed reusable menstrual materials during last menstrual period:	*N* = 166	*N* = 214	
With soap and water	160 (96)	211 (99)	2 (−3, 7)
With water only	6 (4)	3 (1)	
Dried reusable menstrual materials during last menstrual period:	*N* = 166	*N* = 214	
In sunlight	35 (21)	128 (60)	36 (29, 42)
In hiding place	131 (79)	86 (40)	
Location of storing reusable menstrual materials for next use during last menstrual period:			
In a hidden place	116 (70)	105 (49)	
Normally like other clothes	39 (24)	101 (47)	23 (15, 32)
Experienced leakage or staining on outer garments during last menstrual period	*N* = 404 176 (44)	*N* = 382 122 (32)	−12 (−22, −1)

^†^Other than waste bin: disposed openly, in the bush, in the toilet pan, in the canal/open drain

Schoolgirls’ capacity to manage menstruation improved after the intervention, which was evident as an increase in reported drying reusable menstrual materials in the sunlight (PD: 36%; 95% CI: 29, 42). In addition, schoolgirls were more likely to store their reusable menstrual cloth with other clothes for next use (PD: 23%; 95% CI: 15, 32). Fewer girls reported experiences of leakage or blood staining on outer garments during their last menstruation at endline compared to baseline (PD: − 12%; 95% CI: − 22, − 1).

Schoolgirls changed their menstrual materials more frequently during their last menstrual period increasing from a mean of 3.4 times/day at baseline to 4.2 times/day at endline. More schoolgirls disposed of the disposable pad in waste bins or chute disposal system during their last menstrual period (62% vs 55%, PD: 8 (1, 14)) compared to baseline (Table 5).

### Girls’ perceptions of their environment, practices, and comfort

Girls reported higher satisfaction with their menstrual materials during their most recent period at endline compared to baseline, with 59% reporting “satisfied” at endline, compared to 46% at baseline (*p* < 0.005). Similarly, more girls thought school facilities were adequate for their menstrual needs at endline compared to baseline (54% at endline compared to 8% at baseline, *p* < 0.001). At endline, girls reported feeling less anxiety, with 64% disagreeing or strongly disagreeing that they felt anxious at school due to menstruation, compared to 33% at baseline (*p* < 0.001). Girls reported feeling less distraction related to menstruation, with 65% disagreeing or strongly disagreeing that they felt distracted or had trouble concentrating in class at endline, compared to 41% at baseline (*p* < 0.001) (Table 6).

**Table 6:** Girls’ perceptions of their environment, practices, and comfort before and after the intervention

Indicators	Baseline *N* = 404	Endline *N* = 382	Chi-square test for linear trend and ***p***-value
Girls’ satisfaction with the menstrual materials they used during their most recent period			
Satisfied	186 (46)	227 (59)	
Somewhat satisfied	169 (42)	118 (31)	9.81, *p* = 0.00174
Indifferent	38 (9)	29 (8)	
Somewhat unsatisfied	7 (2)	7 (2)	
Unsatisfied	4 (1)	1 (0.3)	
Girls’ perception of the adequacy of school facilities for menstruation management			
Adequate	33 (8)	205 (54)	
Somewhat adequate	211 (52)	135 (35)	167.80, *P* < 0.00001
Neither adequate nor inadequate	59 (15)	25 (7)	
Somewhat inadequate	62 (15)	14 (4)	
Very inadequate	39 (10)	3 (1)	
Girls felt anxious at school due to menstruation during their most recent period			
Strongly agree	47 (12)	13 (3)	
Agree	90 (22)	88 (23)	26.34, *p* < 0.00001
Neither agree nor disagree	134 (33)	74 (19)	
Disagree	80 (20)	146 (48)	
Strongly disagree	53 (13)	61 (16)	
Girls felt distracted or had trouble concentrating in class during most recent period			
Strongly agree	36 (9)	8 (2)	
Agree	93 (23)	57 (15)	26.92, *p* < 0.00001
Neither agree nor disagree	113 (28)	68 (18)	
Disagree	111 (28)	184 (48)	
Strongly disagree	51 (13)	65 (17)	
Most recent period affected girls’ ability to participate in class			
Strongly agree	30 (7)	9 (2)	
Agree	98 (24)	68 (18)	34.25, *p* < 0.00001
Neither agree nor disagree	105 (26)	50 (13)	
Disagree	123 (31)	188 (50)	
Strongly disagree	48 (12)	67 (18)	
Girls feared they might be teased because of menstruation at school during most recent period			
Strongly agree	36 (9)	17 (5)	
Agree	71 (18)	105 (28)	0.005, *p* = 0.94
Neither agree nor disagree	78 (19)	48 (13)	
Disagree	138 (34)	139 (36)	
Strongly disagree	81 (20)	73 (19)	
Girls felt it is common in their school for students to tease girls about menstruation			
Strongly agree	40 (8)	30 (6)	
Agree	112 (21)	118 (22)	3.20, *p* = 0.073
Neither agree nor disagree	110 (21)	75 (14)	
Disagree	158 (30)	180 (34)	
Strongly disagree	107 (20)	125 (24)	
Girls felt comfortable at school during most recent period			
Strongly agree	36 (9)	17 (5)	
Agree	71 (18)	105 (28)	0.75, *p* = 0.386
Neither agree nor disagree	140 (35)	79 (21)	
Disagree	87 (22)	70 (18)	
Strongly disagree	27 (7)	23 (6)	

### Absenteeism

Girls reported absenteeism during their last menstrual period less commonly at endline (20%) compared with baseline (28%) (PD: − 8%; 95% CI: − 14, − 2) (Table 7). However, the number of days missed by schoolgirls during the last menstrual period did not change. The most common reason for school absence during the last menstrual period was experiencing menstrual symptoms. At baseline, 68% of schoolgirls reported that their fellow friends missed school in the last 3 months due to menstruation, which was less common at endline (39%), (PD: -29; 95% CI: − 36, − 23).

**Table 7:** Self-reported school absence due to menstruation at baseline and endline from four urban and rural schools of Bangladesh, 2018

Indicators	Baseline n (%)	Endline n (%)	Prevalence difference (CI)
Girls who missed school days during last menstrual period due to menstruation (Reported)	*N* = 404 111 (28)	*N* = 382 76 (20)	−8 (−13, − 2)
Number of school days missed during last menstrual period because of menstruation (mean, SD)	*N* = 404 0.023 (0)	*N* = 382 0.017 (0)	−0.15 (− 0.31, 0.02)
Menstruation-related reason for missing school during last menstrual period:			
Menstrual symptoms (cramps, pain)	74 (67)	52 (68)	2 (−6, 9)
Do not feel comfortable	32 (29)	14 (18)	−11 (−21, 0)
Excessive bleeding	27 (24)	18 (24)	−1 (− 14, 13)
Afraid of visible menstrual leaks	16 (14)	9 (12)	−3 (−13, 8)
Unavailability of suitable transport during menstruation	6 (6)	1 (1)	−5 (−11, 1)
No available menstrual materials	2 (2)	0	−2 (−5, 1)
School has no menstrual hygiene facilities (no place to change, no water, no soap)	2 (2)	3 (4)	2 (−2, 6)
Number of regular classes (except exam) missed due to menstruation during the last menstrual period (Mean, SD)	*N* = 22 3.7 (2.0)	*N* = 12 2.8 (1.9)	−0.9 (−2.4, 0.5)
Fellow friends missed school in the last 3 months due to menstruation	*N* = 382 259 (68)	*N* = 390 150 (39)	−28 (−34, − 21)

## Discussion

We developed the school-based puberty education sessions in-line with the current national education curriculum, considering context-specific barriers and experiences of girls through an iterative process which was accepted by schoolgirls. Girls used reusable cloth pads instead of the most common prior practice of using scrap cloth both inside and outside their homes which indicates uptake of our intervention. These findings suggest that reusable cloth pads may be an appropriate product to meet the demand for effectively and hygienically managing menstruation. Reusable cloth pads can offer an alternative for cases where other products are not available, desirable, or fit for the purpose [16, 17].

Knowledge scores significantly improved among schoolgirls suggesting that course content was delivered in a manner understandable to students. According to a recent study, girls’ menstrual experiences at school were shaped by common language and education around menstruation, as well as encouragement from teachers and peers, which led to their ease and confidence in managing menstruation [18]. Centred on the local context, we combined the software intervention (school sessions, education booklets) with the hardware intervention (menstrual absorbents and disposal facilities). Sound knowledge and education concerning menstruation were essential components, as suggested by girls, in order to understand the mechanisms of their own bodies, obtain information about effective and hygienic practices and eliminate negative stereotypes and stigma [19]. Schoolgirls also needed clean materials that were adequately absorbent for menses, facilities in secured areas for changing menstruation materials, as well as access to water, soap, and appropriate locations for washing reusable materials and their own bodies [16].

Other potential explanations for the uptake of reusable cloth were that the cloth pad could be dried in an open location in sunlight (as it was red in colour and looks like a handkerchief which makes it difficult to identify as a menstrual cloth) and prevented stains on outer garments due to a water-resistant bottom layer, similar to findings from Shah et al. 2013. However, since reusable cloth pads require sufficient water to wash, this level of maintenance may be difficult to maintain. Cloth pad cleanliness might be compromised in populations where water supply is highly constrained, including where water is unavailable in the toilet area when changing, especially when women and girls are in public places such as in school [20, 21] or in a slum [22–24].

Schoolgirls in our program were more likely to store their used reusable menstrual materials with their regular clothes for use during the next menstrual cycle at endline compared to baseline. Other studies suggest that girls usually stored menstrual cloth in dirtier places between periods [5, 8]. This finding suggests that with the provision of adequate support, a significant proportion of girls may be able to use a reusable pad safely (and with more long-term support this proportion may be increased,) making it a viable intervention to investigate further.

We provided a chute disposal system, which provided functional disposal infrastructure to support the safe disposal of single-use menstrual pads without affecting the environment through open disposal [21]. Available local sanitation masons from the water, sanitation, and hygiene sector can be brought in to construct safe disposal facilities. Other than appropriate menstrual materials, adequate WASH facilities and supportive resources are also crucial enablers for girls [16]. Due to stigma associated with menstruation, women and girls have emphasized the importance of a secure and private location for comfortably changing, disposing, or washing products [11, 25].

Schoolgirls felt less anxious at school due to menstruation and fewer felt that their period affected their ability to participate in class, which suggested that the intervention created a more supportive environment for them at school. However, fear of teasing increased slightly from baseline to endline, possibly due to intervention materials seen by the boys. This stands in contrasts to positive results reported in the recent MENISCUS study conducted in two secondary schools in Entebbe, Uganda [26]. These results raise questions about how best to engage boys in future MHM interventions in Bangladesh.

There was some change in reported absenteeism due to menstruation from baseline to endline. This must be interpreted with caution, especially as respondents reported their own absenteeism due to menstruation at half the frequency they reported for their friends. The alternative of documenting school attendance is often difficult due to incomplete or inaccurate school registers [27].

### Strengths and limitations

Our intervention was developed based on formative research following an iterative process to develop a context-specific and appropriate intervention [28]. The high uptake of the intervention indicated the primary success of the process. We conducted our study in a small number of schools, so the encouraging uptake may not represent how well this intervention would work across a more diverse set of schools. We conducted a before-after study to evaluate the pilot intervention. This approach risks overestimating the impact of interventions in any dynamic community as there could be other changes occurring more generally in attitudes towards menstruation around the country [22]. A randomized controlled trial could provide an unbiased estimate of the impact of the intervention.

We measured the self-reported school absenteeism during most recent menstrual period which may introduce reporting bias; other measures like daily diary or fingerprint sensor could be used in the future studies. We selected two independent groups of respondents during for pre and post intervention assessments to reduce selection/assignment bias [29]; we recommend that future randomized controlled trials avoid this bias. We did not assess the cleanliness of toilets, which may have impacted toilet use during menstruation and school absence. Girls often feel disgusted and do not want to use toilets when they are not adequately maintained [30]. Future interventions that include strategies to maintain clean toilets may generate improved outcomes. We did not use standard tools such as the Menstrual Practice Needs Scale [31] because it was not available at the time of our data collection.

## Conclusion and recommendation

There is a controversy regarding the impact of MHM interventions on attendance and schoolgirl health. Interventions are most likely to generate benefits if they are well designed for the context. This study showed that a carefully developed intervention engaging various stakeholders and including essential equipment, supplies and educational materials can be implemented in Bangladesh. A controlled evaluation of whether such thoroughly designed interventions reduce absenteeism among schoolgirls would provide information that could guide policy in both government and private schools.

## Supplementary Information

**Additional file 1: Supplementary Table 1.** Description of the intervention package.

**Additional file 2.**

## Data Availability

The datasets used and/or analysed during the current study are available from the corresponding author on reasonable request.

## References

[1] Hennegan J (2019). Women’s and girls’experiences of menstruation in low- and middle-income countries: a systematic review and qualitative metasynthesis. Plos Med.

[2] JMP (2012). Meeting Report of JMP Post-2015 Global Monitoring Working Group on Hygiene.

[3] unicef (2019). Guidance on Menstrual Health and Hygiene.

[4] WEDC (2012). Developing knowledge and capacity in water and sanitation: menstruation hygiene management for schoolgirls in low-income countries.

[5] Alam MU (2017). Menstrual hygiene management among Bangladeshi adolescent schoolgirls and risk factors affecting school absence: results from a cross-sectional survey. BMJ Open..

[6] Sommer M (2016). A time for global action: addressing girls’ menstrual hygiene management needs in schools. Plos Med..

[7] MHMinTen (2014). Meeting Report: MHM in Ten.

[8] Hennegan J, Montgomery P (2016). Do menstrual hygiene management interventions improve education and psychosocial outcomes for women and girls in low and middle income countries?. A systematic review. Plos One..

[9] MinistryofEducation (2015). Circular: to improve the toilet and sanitation conditions in secondary and higher secondary schools, madrasas and technical and vocational institutions.

[10] BBS (2019). Bangladesh National Hygiene Survey 2018.

[11] Jahan F (2020). Piloting an acceptable and feasible menstrual hygiene products disposal system in urban and rural schools in Bangladesh. BMC Public Health.

[12] BCCP, Annual HPN SBCC Monitoring Report 2017-2018. 2018, Bangladesh Center for Communication Programs: Dhaka. Accessed 14 July 2020.

[13] SMC (2020). Social Marketing Company.

[14] WaterAid (2016). School WASH research: Bangladesh country report.

[15] Cochran WG (1954). Some methods for strengthening the common χ<sup>2</sup> tests. Biometrics.

[16] Sommer M, Sahin M (2013). Overcoming the taboo: advancing the global agenda for menstrual hygiene management for schoolgirls. Am J Public Health.

[17] Ellis A (2016). WASH challenges to girls’ menstrual hygiene management in metro Manila, Masbate, and south Central Mindanao, Philippines.

[18] Hennegan J (2017). A qualitative understanding of the effects of reusable sanitary pads and puberty education: implications for future research and practice. Reprod Health.

[19] Chothe V (2014). Students’ perceptions and doubts about menstruation in developing countries: a case study from India. Health Promot Pract.

[20] Sivakami M (2019). Effect of menstruation on girls and their schooling, and facilitators of menstrual hygiene management in schools: surveys in government schools in three states in India, 2015. J Glob Health..

[21] Kaur R, Kaur K, Kaur R (2018). Menstrual hygiene, management, and waste disposal: practices and challenges faced by girls/women of developing countries. J Environ Public Health.

[22] Alam MU (2017). Behaviour change intervention to improve shared toilet maintenance and cleanliness in urban slums of Dhaka: a cluster-randomised controlled trial. Tropical Med Int Health.

[23] Saxton RE (2017). If I do not have enough water, then how could I bring additional water for toilet cleaning?! Addressing water scarcity to promote hygienic use of shared toilets in Dhaka, Bangladesh. Trop Med Int Health.

[24] Yeasmin F (2017). Piloting a low-cost hardware intervention to reduce improper disposal of solid waste in communal toilets in low-income settlements in Dhaka, Bangladesh. BMC Public Health.

[25] Budhathoki SS (2018). Menstrual hygiene management among women and adolescent girls in the aftermath of the earthquake in Nepal. BMC Womens Health.

[26] Miiro G (2018). Menstrual health and school absenteeism among adolescent girls in Uganda (MENISCUS): a feasibility study. BMC Womens Health.

[27] Hennegan J (2020). Measurement in the study of menstrual health and hygiene: a systematic review and audit. Plos One.

[28] Gurley ES (2013). Behaviour change intervention to reduce caregivers’ exposure to patients’ oral and nasal secretions in Bangladesh. Int J Infect Control..

[29] Ho AMH (2018). Bias in before–after studies: narrative overview for anesthesiologists. Anesth Analg.

[30] Coswosk ÉD (2019). Having a toilet is not enough: the limitations in fulfilling the human rights to water and sanitation in a municipal school in Bahia, Brazil. BMC Public Health.

[31] Hennegan J (2020). Measuring menstrual hygiene experience: development and validation of the menstrual practice needs scale (MPNS−36) in Soroti, Uganda. BMJ Open.

