# Supplementary material for: Evaluation of a menstrual hygiene intervention in urban and rural schools in Bangladesh: a pilot study
Source: BMC Public Health. 2022 Jun 2;22:1100. doi: 10.1186/s12889-022-13478-1 (PMC9161596; doi:10.1186/s12889-022-13478-1)
Supplement: Supplementary file 1 — Additional file 1: Supplementary Table 1. Description of the intervention package. [file 12889_2022_13478_MOESM1_ESM.docx]

## Supplementary Table 1: Description of the intervention package

| **MHM Working Group**  The MHM Working Group was formed as a component of the study with the aim to create a platform of all Government and non-government stakeholders working to improve MHM in Bangladesh, both at schools and in the community. The intention of the working group was to involve all the stakeholders in developing a combination of interventions and targeted menstrual hygiene education to have a positive impact on girls’ school attendance, academic performance, and overall educational experience and in the long run, motivate educators and policymakers in Bangladesh to implement a nationwide menstrual health management strategy. The Working Group includes stakeholders from the Ministry of Education, Ministry of Health and Family Welfare (MOH&FW), Directorate General of Health Services (DGHS), Directorate of Primary Education (DPE), Directorate of Secondary and Higher Education, Directorate of Madrassa Education, Education Engineering Department, Department of Public Health Engineering, Directorate of Technical Education, representatives from BSMMU, corporations and NGOs working on menstrual hygiene issues as active members. The major roles of this Working Group were:  1. Overseeing and supporting project activities, recommendation on intervention decisions, and components  2. Linking different stakeholders in the MHM field  3. Guiding for facilitating policy implementations and advocacy based on study findings  4. Guiding to develop MHM action/implementation plan and support to further scale up  Four MHM working group meetings were held successfully in different venues (icddr,b office, Shornokishoree Network Foundation office, and Directorate of Secondary and Higher Education). |
| --- |
| **Puberty Education Curriculum: "*Know Yourself & Grow*"**  The puberty education curriculum included one teachers' guide and one flip chart or PowerPoint presentation for students, titled "Know Yourself & Grow". The contents of the curriculum included introduction to puberty, physical changes, mental and emotional changes, reproductive systems, nocturnal emissions (or wet dreams), menstrual cycle, menstrual hygiene management, supporting our peers, problem-solving, overcoming stigma and nutrition for growing adolescents. Headteachers and selected other teachers were trained to prepare them to provide the sessions in class. We provided each of the selected teachers with an easel for the flipcharts. |
| **MHM Packs**  The pack included low-cost (US$0.7) cloth pads, underwear, plastic bag (to store/take the stained cloth pad back to home for washing/drying) and menstruation tracking calendars. Each school also had an "adolescent corner", in which girls had access to disposable sanitary pads, underwear, and a registrar book for tracking to manage sudden menstruation under supervision of a janitor and class cabinet members. We initially gave out 10 packets [(10x8) = 80 pieces] of disposable pads to each school and then 5 packets [(5x8) =40 pieces] when the stocks ran out. We provided 10 sets of underwear to each school. |
| **Improvements to school sanitation facilities**  Based on the formative research findings, we renovated school toilets (e.g. basin, light bulb, door, lock, and others) to make them girl-friendly. The improvement to the sanitation facilities included constructing a chute disposal system in one girls' bathroom in each school and other minor changes such as fixing door locks, lights, and other minor improvements where needed. |
| **School gender committees**  We formed a gender committee in each school consisting of students (both boys and girls) of grade 5-10, head and trained teachers, janitors, School Management Committee members, parents, Education Officers and Upazila Nirbahi Officer. The committee members were to hold monthly meetings, discuss puberty and menstrual hygiene issues as well as about the maintenance of MHM intervention activities. After each meeting, the boys were to discuss puberty issues with three other boys and girls were to discuss menstruation with three other girls, so that these issues would be disseminated among the students. |
