# Supplementary material for: Evaluation of a menstrual hygiene intervention in urban and rural schools in Bangladesh: a pilot study
Source: BMC Public Health. 2022 Jun 2;22:1100. doi: 10.1186/s12889-022-13478-1 (PMC9161596; doi:10.1186/s12889-022-13478-1)
Supplement: Supplementary file 2 — Additional file 2. [file 12889_2022_13478_MOESM2_ESM.docx]

**School students (Girls) survey of menstrual hygiene management interventions among elementary and schools in rural Bangladesh**

**†KejgvÎ QvÎx‡`i Rb¨ [Only for female Students]**

**†mKkb-1 (cÖkœvejx mbv³KiY I DËi`vZv msµvšÍ Z_¨)**

**Section 1 (Questionnaire identification and information about respondent)**

- 1. cÖkœcÎ mbv³Kvix bv¤^vi [Questionnaire identification number]..............
  2. ÷vwW GjvKv [StudyArea].................................................................

MÖvg [Rural] 0

kni [Urban] 1

- 1. ¯‹z‡ji bvg [School name].....................................................................
  2. Avcwb †Kvb †kªbx‡Z c‡ob? (K¬vm Abyhvqx 5 ‡_‡K10 †KvW e¨envi Kiæb) [Which class do you read? (Use code 5 to 10)].............................................................................................................
  3. kvLv [Section].........................................
  4. µwgK bs [Roll]............................

107.a. Avcwb KZ mgq a‡i GB ¯‹z‡j co‡Qb? (DËi gv‡m wjLyb) [How long do you read in that school? (Answer write down in month] .............................................

- 1. Z_¨ msMÖ‡ni ZvwiL [Date of interview]...................................................//
  2. FRA-Gi bvg Ges AvBwW [FRA name & ID].............................................

**DËi`vZv msµvšÍ Z_¨ [Respondent’s information]**

- 1. DËi`vZvi bvg [Name of respondent]: _______________________
  2. DËi`vZvi eqm [Age of respondent:

eQi [Year]: 

gvm [Month]: 

- 1. DËi`vZvi gv †Kvb K¬vm ch©šÍ cov‡jLv K‡i‡Qb? (hw` DËi`vZv ej‡Z bv cv‡i, Zvn‡j Ó99Ó †KvW e¨envi Kiæb) [Education of mother of the respondent (If respondent says don’t know then use code “99”)]......
  2. DËi`vZvi evev †Kvb K¬vm ch©šÍ cov‡jLv K‡i‡Qb? (hw` DËi`vZv ej‡Z bv cv‡i, Zvn‡j Ó99Ó †KvW e¨envi Kiæb) [Education of father of the respondent(If respondent says don’t know then use code “99”)]..........................................................................................................................
  3. DËi`vZvi evevi cªavb †ckv [Main occupation of father of the respondent]
  4. DËi`vZvi gv‡qicªavb †ckv [Main occupation of mother of the respondent]

| **‡ckv †KvW [Occupation Code]:**  01.N‡i KvR K‡i/M„wnYx [Homemaker for own HH/Housewife]  02.kªwgK (KvwqK kªg cÖ`vb K‡i) [Labor (physical labor)]  03.‡eZbfy³ Kg©Pvix [Salaried job]  04.wg¯¿x (ivRwgw¯¿, myZvi/ KvVwgw¯¿, ˆe`¨ywZK wgw¯¿, m¨vwbUvwi wg¯¿x BZ¨vw`) [Mason/Carpenter/Electrician/Plumber]  05.f¨vb/wiKkv PvjK/e¨vUvwi PvwjZ A‡Uvwi·v PvjK [Van/Rickshaw puller/battery driven auto riksha driver]  06.gywP [Cobbler/maker]  07. ‡`vKvb`vi/e¨emvqx/†dwiIqvjv [Shopkeeper/Business/ambulent vendor/ pharmacy]  08.`wR©(A‡_©i wewbg‡q wbR N‡i ev †`vKv‡b †mjvB K‡i _v‡K) [Tailor (both in home and shop)] | 9.WªvBfvi[Driver]  10.KzwUi wkí [Cottage industry]  11. †cvkvK KviLvbvi kªwgK [Garment worker]  12.Kv‡Ri †jvK [Domestic maid /servant]  13.wf¶zK [Begger]  14.‡eKvi/ A¶g [Unemployed/ Disabled]  15.g„Z/wb‡LuvR [Died/untraced]  16. we‡`‡k _v‡K [Staying abroad]  17. Wv³vi/DwKj/ BwÄwbqvi [Doctor/ lawyer/engineer]  18. K…lK [Farmer]  19. MÖvg¨/‡nvwgI Wv³vi [Village doctor/Homio doctor]  77.Ab¨vb¨ (eY©bv wjLyb) [Others (specify)]______________  99.Rvwbbv [Don’t know] |
| --- | --- |

- 1. Avcbvi wK Avcb eo fvB Av‡Q? [Do you have any older brothers in your household?]..............

bv [No]................................. 0

n¨uv [Yes]............................... 1

- 1. Avcbvi wK Avcb eo ‡evb Av‡Q? [Do you have any older sisters in your household?]……….

bv [No]................................. 0

n¨uv [Yes]............................... 1

**†mKkb-2: eqtmwÜKvj-gvwmK msµvšÍ aviYv Ges cwi®‹vi-cwi”QbœZv PP©v**

**[Section 2: Perception about adolescence, menstruation and hygiene practice during menstruation]**

- 1. hLb GKwU †g‡q wK‡kvix †_‡K mvevwjKv nq ZLb Zvi wK wK ai‡bi cwieZ©b n‡q _v‡K? **(GKvwaK DËi MÖnb‡hvM¨)?** [What happens to a girl’s as she changes from a girl to an adult during adolescent period?] **(multiple answers allowed here)**……….................. ….

¯Íb eo nq [Breasts develop]............................................................ 1

wncm ev‡o [Hips widen]................................................................... 2

gvwmK nq [Menstruation begins]....................................................... 3

gvbwmK cwieZ©b nq [Emotional changes]............................................ 4

kix‡ii wewfbœ As‡k (eyK, eMj, †hŠbv½, evû Ges cv) ‡jvg MRvq [Pubic hair grows]...... 5

D”PZv e„w× cvq [Height increases]............................................................................6

kixi Mf©avi‡bi Rb¨ mÿg nq [The body prepares to be

able to conceive a baby]................................................................... 7

‡hvbx c_ w`‡q i³cvZ nq [Blood exits via the vagina]................................ 8

Mjvi ¯^i cwieZ©b nq [Change voice] .......................................................... 9

Ab¨vb¨ (wbw`©ó K‡i wjLyb).................................................................................................77

Rvwb bv [DK]............................................................................................ 99

- 1. Avcwb Rv‡bb wK, Avgv‡`i †`‡ki (evsjv‡`‡ki) †g‡q‡`i Av`¨/cÖ_g gvwm‡Ki Mo eqm KZ? [Do you know, what is the average age of a Bangladeshi girl’s first period/menarche?]

eQi [Year]: 

gvm [Month]: 

Rvwbbv [Don’t know]:

- 1. gvwm‡Ki mgq, cÖwZ gv‡m wK Kvi‡b i³cvZ nq? [During menstruation, what is the reason or purpose for the monthly bleeding?]..................................................................................

**DËi ¸‡jv c‡o †kvbvb (Response options should be read)**

kixi †_‡K `~wlZ/welv³ c`v_© cwi®‹vi Ki‡Z [To cleanse toxins from the body].... 1

kixi †_‡K Rivqyi cyivZb c`v©wU †ei n‡q Av‡m hv‡Z bZzb c`v© MwVZ n‡Z cv‡i

[To pass the old lining of the uterus from the body so a fresh lining can be formed]........................................................................................................ 2

‡g‡q‡`i cÖRbb A½¸wj‡ZGKwU mgm¨v Av‡Q Zv wb‡`©k Ki‡Z [To indicate that there is a problem with the girl’s reproductive organs].............................................. 3

‡g‡q‡`i Amy¯’Zv ‡evSv‡Z [To indicate that the girl is sick].................................. 4

Ab¨vb¨ (wbw`©ó K‡i wjLyb)...................................................................................................... 77

Rvwb bv [DK].......................................................................................................................... 99

- 1. mvavibZ KZ w`b ci ci ‡g‡q‡`i gvwmK nq? [Some girls and women have longer menstruation cycle, others have shorter menstruation cycle. On **average**, how often does a girl or woman typically get a period (in other words, how long is the entire cycle)?]

w`b [days]

Rvwb bv [DK]...... 99

- 1. †Kvb ‡Kvb †g‡q‡`i †ewkw`b a‡i gvwm‡Ki i³cvZ nq Avevi Kv‡iv Kgw`b a‡i nq, M‡o cÖwZ gvwm‡K KZ w`b i³cvZ nq? [Some girls and women have longer periods of bleeding, others have shorter periods of bleeding. On **average**, how long is each period of bleeding?]

**DËi ¸‡jv c‡o †kvbvb (Response options should be read)**

1 w`b †_‡K 3 w`b [1-3 days]............................ 1

3 w`b †_‡K 7 w`b [3-7 days]............................ 2

7 w`b †_‡K 10 w`b [7-10 days]...................... 3

10 w`‡bi ‡ewk [More than 10 days]............ 4

Rvwb bv [DK]……………………………………….... 99

- 1. Avcbvi Rb¥ ZvwiL KZ? [What is your date of birth?].....................//
  2. Avcbvi wK gvwmK n‡q‡Q? [Have you experienced with menstruation yet?].........................

bv [No]................................. 0

n¨uv [Yes]............................... 1

***Skip Note: hw` 208Gi DËi 0 nq, Z‡e 239, 406, 407, 412, 413,414 , 415, 416, 417, 418, 502, 503, 504, 505b, 510, 516, 517, 518, 519, 520, 521, 522, 523, 524, 525, 527, 527a, 527b, 527c, 527d,528, 529, 530, 531, 532*** wRÁvmv Kiæb

***[If answer of 208 is 0, then ask to 239, 406, 407, 412, 413, 414, 415, 416, 417, 418, 502, 503, 504, 505b, 510, 516, 517, 518, 519, 520, 521, 522, 523, 524, 525, 527, 527a, 527b, 527c, 527d, 528, 529, 530, 531, 532]***

- 1. hLb cÖ_g gvwmK ïiæ n‡qwQj ZLb Avcbvi eqm KZ wQj? (g‡b Ki‡Z bv cvi‡j 99 wjLyb) [How old were you when you started menstruating, insert 99 if cannot remember?]...........................

eQi [Year]:

gvm [Month]:

- 1. GB ch©šÍ Avcbvi KZevi gvwmK n‡q‡Q? (hw` g‡b Ki‡Z bv cv‡i Zvn‡j 99 †KvW Kiæb) [How many menstrual periods have you had in total? [Insert 99 if cannot remember]
  2. `qv K‡i Avcbvi me©‡kl gvwmK ïiæi ZvwiLUv GKUz ej‡eb? [Would you please tell me the date of your last started menstruation?]............................................................. //
  3. ~~Dropped~~
  4. hLb Avcbvi cÖ_g gvwmK n‡qwQj ZLb GUv Avcwb Kv‡K e‡jwQ‡jb? [When you first got your period, whom did you tell?]

gv [Mother]............................................................................ 1

`v`x/bvbx [Grand-mother]....................................................... 2

evev [Father]............................................................................ 3

‡evb/fvex[Sister/Sister-in law]................................................ 4

fvB[Brother]........................................................................... 5

AvZ¥xq-gwnjv[Relative-female]............................................. 6

AvZ¥xq-cyiæl[Relative-male].................................................. 7

eÜ z[Friend]............................................................................. 8

cÖwZ‡ekx [Neighbors].............................................................. 9

KvD‡K ewjwb [Nobody]......................................................... 10

Avwg KvD‡K ewjwb, Ab¨ †KD Avgv‡K †`‡L civgk© cÖ`vb K‡iwQj

[Someone noticed and advised without me asking]...............11

g‡b bvB [Don’t remember]...................................................12

Ab¨vb¨ (wbw`©ó K‡i wjLyb)[Other (specify)].................................. 77

- 1. hw` DËi 11 nq Zvn‡j, †K civgk© cÖ`vb K‡iwQj ? [If the answer is 11, who was that?]

gv [Mother]............................................................................ 1

`v`x/bvbx [Grand-mother]......................................................... 2

evev [Father]............................................................................ 3

‡evb/fvex[Sister/Sister-in law]................................................ 4

fvB[Brother]........................................................................... 5

AvZ¥xq-gwnjv[Relative-female].............................................. 6

AvZ¥xq-cyiæl[Relative-male]................................................ 7

eÜz[Friend]............................................................................. 8

cÖwZ‡ekx [Neighbors].............................................................. 9

Ab¨vb¨ (wbw`©ó K‡i wjLyb)[Other (specify)].................................. 777

- 1. me©‡kl gvwm‡Ki mgq evwo‡Z _vKv Ae¯’vq Avcwb wK wK e¨envi K‡iwQ‡jb **(GKvwaK DËi MÖnb‡hvM¨)**? [What did you use during your last menstruation when you are inside home?] (multiple answers allowed here)..........................................................................

cyivZb Kvco (Kvc‡oi UzKiv)[Old Cloth (rag)] ................... 1

cybe©¨envi ‡hvM¨ c¨vW [Reusable Manufactured Sanitary Pads]........ 2

Mv‡g©‡›Um Gi e¨eüZ Kvc‡oi UzKiv [Jute of garments]............... 3

bZzb Kvco[New cloth]........................................................... 4

m¨vwbUvix c¨vW (cybe©¨envi ‡hvM¨ bq) [Disposable Sanitary Pads]...... 5

U¨v¤úyb [Tampons]......... .......................................................... 6

gvwmK Gi Rb¨ e¨eüZ Kvc[Menstrual cup]............................ 7

¯cÄ[Sponges]...................................................................... 8

Zzjv [Cotton/wool].................................................................. 9

wUmy¨ †ccvi [Tissue paper]...................................................... 10

‡Kvb wKQzBbv [Nothing]............................................................. 11

AvBwmwWwWAviwe n‡Z cÖvß Kvco [Cloth pad provided by icddr,b] ...... 12

ïaygvÎ AvÛviIq¨vi/c¨vw›U [Only underwear] ........................................ 13

ej‡Z ivwR bv [Refused to say] ............................................... 66

Ab¨vb¨ (wjLyb)[Other: Specify:] ............................................. 77

- 1. me©‡kl gvwm‡Ki mgq hLb Avcwb evwoi evB‡i wQ‡jb ZLb Avcwb wK wK e¨envi K‡iwQ‡jb **(GKvwaK DËi MÖnb‡hvM¨)**? [What did you use during your last menstruation while out of home **(multiple answers allowed here)**?]] ............................................................

cyivZb Kvco (Kvc‡oi UzKiv)[Old Cloth (rag)] ................... 1

cybe©¨envi ‡hvM¨ c¨vW [Reusable Manufactured Sanitary Pads]........ 2

Mv‡g©‡›Um Gi e¨eüZ Kvc‡oi UzKiv [Jute of garments]............... 3

bZzb Kvco[New cloth]...................................................... 4

m¨vwbUvix c¨vW (cybe©¨envi ‡hvM¨ bq)[Disposable Sanitary Pads]...... 5

U¨v¤úyb [Tampons]......... ..........................................................6

gvwmK Gi Rb¨ e¨eüZ Kvc[Menstrual cup]............................ 7

¯cÄ[Sponges]...................................................................... 8

Zzjv [Cotton/wool].................................................................. 9

wUmy¨ †ccvi [Tissue paper]...................................................... 10

‡Kvb wKQzBbv [Nothing]............................................................. 11

AvBwmwWwWAviwe n‡Z cÖvß Kvco [Cloth pad provided by icddr,b] ...... 12

ïaygvÎ AvÛviIq¨vi/c¨vw›U [Only underware] ................................ 13

ej‡Z ivwR bv [Refused to say] ............................................... 66

Ab¨vb¨ (wjLyb)[Other: Specify:] ............................................. 77

cÖ‡hvR¨ bq [Not applicable]...................................................... 88

- 1. me©‡kl gvwmK PjvKvjxb mgq KZ Nb Nb Avcwb Avcbvi cÖmv‡ei RvqMv/gvwm‡Ki iv¯Ív (†hŠbv½) fvjfv‡e cwi®‹vi K‡iwQ‡jb? [During your most recent period, how frequently you thoroughly wash your genital area when you are menstruating?]

w`‡b GKevi [Once per day] ................................................. 1

w`‡b GKev‡ii †ewk[More than one per day]............................. 2

ayB bv [Don’t wash]............................................................... 3

Ab¨vb¨ (wjLyb)[Other: Specify:] ............................................. 77

***Skip Note: hw` 216& 217Gi DËi 11/66 nq, Z‡e 234G P‡j hvb***

***[If the answer of 216& 217 is 11/66, then skip to 234]***

- 1. me©‡kl gvwmK PjvKvjxb mg‡q Avcwb KZ evi Avcbvi gvwm‡Ki e¨eüZ `ªe¨wU cwieZ©b K‡iwQ‡jb? (ej‡Z ivwR bv n‡j 66 wjLyb) [During your most recent period, how many times did you typically change the menstrual product used for menstruation?(66=refused to say)]

A. mKvj †_‡K mÜ¨v [From morning to evening]

B. mÜ¨v †_‡K mKvj [From evening to morning]

219.a. me©‡kl gvwmK PjvKvjxb mg‡q Avcwb ‡Kv_vq Avcbvi e¨eüZ gvwm‡Ki `ªe¨wU cwieZ©b K‡iwQ‡jb **(GKvwaK DËi MÖnb‡hvM¨)**? [During your most recent period, where did you typically change the menstrual product? **(multiple answers allowed here)**]

cvqLvbvq [In toilet]

‡MvmjLvbvq [In bathroom]

wb‡R‡`i K‡ÿ [In main room]

Avjv`v cwieZ©b K‡ÿ [Seperate changing room]

777. Ab¨vb¨ (wjLyb) [Others]

219.b. me©‡kl gvwmK PjvKvjxb mg‡q Avcwb hLb Avcbvi e¨eüZ gvwm‡Ki `ªe¨wU cwieZ©b K‡iwQ‡jb ZLb Avcwb GUv †`‡L wK DwØMœ/`ywðšÍv MÖ¯’ n‡qwQ‡jb? [During your most recent period, did you worry about being observed while changing the menstrual product?]

bv [No]................................. 0

n¨uv[Yes]............................. 1

Rvwb bv[DK]........................... 99

- 1. me©‡kl gvwm‡Kimg‡q **m‡ev©”P** KZ N›Uv Avcwb gvwm‡Ki e¨eüZ `ªe¨wUe¨envi K‡iwQ‡jb? [During your most recent period, what was the LONGEST time you went without changing your pad/cloth?]

N›Uv [hours]

- 1. me©‡kl gvwm‡Ki mgq hw` Avcwb cybe©¨envi ‡hvM¨ bq Ggb gvwm‡Ki `ªe¨ e¨envi K‡i _v‡Kb, Zvn‡j Avcwb GwU †Kv_vq †d‡jwQ‡jb? **(GKvwaK DËi MÖnb‡hvM¨)** [During your most recent period, if you used a disposable sanitary product, in what ways did you dispose of it? **(Check all that apply)**]

Db¥y³ ¯’v‡b ‡d‡j ‡`B [Openly disposed]... 1

‡SvcSv‡o †d‡j w`B [In the bush] ...... 2

Uq‡j‡Ui c¨v‡bi wfZ‡i †dwj [In toilet pan].......3

Lvj/wej/‡Wvev/bvjv †d‡j w`B [In the canal] ..........................4

Wv÷we‡b †d‡j w`B[In waste bin].............. 5

cywo‡q †dwj [Burned/incinerate]....................6

‡Lvjv †Wªb [Open drain]...............................7

gvwU‡Z cyu‡Z †dwj [Burried] ........................ 8

Ab¨vb¨ (wjLyb) [Other (Specify)]................................77

cÖ‡hvR¨ bq [Not applicable]........................................ 88

- 1. hw` DËi`vZv cybe©¨envi ‡hvM¨ `ªe¨ e¨envi K‡i Zvn‡j,Avcwb wK w`‡q KvcowU cwi®‹vi K‡ib? (GKvwaK DËi MÖnb‡hvM¨) [If you used reusable product, how did you clean/ wash this cloth (rag) during last menstrual period?] **(mark all that apply)**?]..............................

mvevb/wWUvi‡R›U Ges ¯^vfvweK/VvÛv cvwb w`‡q [With soap and normal/cold water]... 1

mvevb I Mig cvwb w`‡q[With soap & hot water].............................................. 2

m¨vfjb/‡WUj Ges ¯^vfvweK/VvÛv cvwb w`‡q [With savlon/ detol and normal/cold].............................................................................................. 3

m¨vfjb/‡WUj I Mig cvwb w`‡q[With savlon/ detol & hot water]....................... 4

mvevb,m¨vfjb/‡WUj I VvÛv cvwb w`‡q [With soap, savlon/ detol &cold water]...... 5

mvevb,m¨vfjb/‡WUj I Mig cvwbw`‡q [With soap, savlon/ detol & hot water]... 6

Mig cvwb w`‡q [With hot water]....................................................................... 7

ïay cvwb w`‡q [With only water]....................................................................... 8

cwi®‹vi K‡ib bv [Don’t clean]........................................................................ 9

Ab¨vb¨ (wjLyb)[Other (Specify)]..........................................................................77

cÖ‡hvR¨ bq [Not applicable].......................................................................... 88

***Skip Note: hw`222Gi DËi 9/88 nq, Z‡e 230G P‡j hvb***

***[If answer of 222is 9/88, then skip to 230]***

- 1. me©‡kl gvwm‡Ki mgq Avcwb ‡Kv_vq KvcowU cwi®‹vi K‡iwQ‡jb? (GKvwaK DËi MÖnb‡hvM¨) [Where didyou clean/ wash this cloth during your last period? **(mark all that apply)**].................

Uq‡j‡U [In toilet]................................................................... 1

‡MvmjLvbvq [In bathroom]....................................................... 2

cvewjK U¨vc [Public tap]......................................................... 3

wUDeI‡q‡j [Under tube well]................................................... 4

wUDeI‡qj/Uq‡j‡Ui cv‡k[Beside the toilet/tubewell]................. 5

cyKyi/b`xi cv‡o [Beside pond/river]........................................ 6

cyKyi/b`x‡Z[In the pond/river]................................................. 7

Avjv`v RvqMvq [In seperate place] ............................................ 8

Ab¨vb¨ (wjLyb)Other (Specify).................................................. 77

Rvwb bv[DK]............................................................................ 99

223.a. me©‡kl gvwmK PjvKvjxb mg‡q Avcwb hLb Avcbvi e¨eüZ gvwm‡Ki `ªe¨wU cwi®‹vi K‡iwQ‡jb ZLb wK Avcwb GUv †`‡L wK DwØMœ/`ywðšÍv MÖ¯’ n‡qwQ‡jb? [During your most recent period, did you worry about being observed while washing the menstrual product?]

bv [No]................................. 0

n¨uv[Yes]............................. 1

Ab¨ †KD cwb®‹vi K‡i †`q [Clean by someone].................... 2

Rvwb bv[DK]........................... 99

- 1. KvcowU cwi®‹vi Kivi Rb¨ Avcwb ‡Kv_v ‡_‡K cvwb ‡c‡q _v‡Kb? **(GKvwaK DËi MÖnb‡hvM¨)** [Where did you get the water for washing/ cleaning this cloth (rag)? **(mark all that apply)]............**.............................................................

AMfxi wUDeI‡qj (250 wd‡Ui Kg) [Shallow tube well(<250 feet)]....... 01

Mfxi wUDeI‡qj (250 wd‡Ui †ekx) [Deep tube well(250+ feet)]............. 02

msiw¶Z Ku~qv [Protected ring/dug well].......................................... 03

Amsiw¶Z K~uqv[Unprotected dug well]........................................... 04

AMfxi Zviv cv¤ú[Shallow Tara pump]............................................... 05

Mfxi Zviv cv¤ú[Deep Tara pump].................................................... 06

N‡ii wfZi U¨vc ev cvB‡ci cvwb [Piped water into dwelling]....................07

DVv‡b U¨vc ev cvB‡ci cvwb [Piped water into yard/plot]........................... 08

cvewjK U¨vc [Public tap/stand pipe]................................................... 09

wdëvi/Av‡m©wbK wdëvi [Arsenic filter ]................................................. 10

Av‡m©wbK †kvabvMvi [Arsenic free treatment plant]................................. 11

msiw¶Z SYv©i cvwb [Water from protected spring]................................12

Amsiw¶Z SYv©i cvwb[Water from unprotected spring]..........................13

e„wói cvwb [Rainwater].................................................................... 14

U¨vsKvi UªvK [Tanker truck]............................................................... 15

†QvU U¨vsKhy³ KvU© [Cart with small tank].............................................. 16

b`x/euva/†jK/cyKzi/†mP bvjv‡_‡K RxevYy-gy³KiY cvwb

[Pathogen treatment plant (Pond Sand Filter)]:

River/dam/lake/ponds/stream/canal/irrigation channel]....................17

b`x/evua/†jK/cyKzi/†mP bvjv‡_‡K mivmwi msM„nxZ cvwb

[Directly from River/dam/lake/ponds/stream/canal/irrigation channel]...18

‡evZjRvZ cvwb [Distilled bottled water]............................................... 19

‡dvUv‡bv cvwb [Boiled water]............................................................. 20

we‡µZv †_‡K cÖvß cvwb [Vandor provided water].................................... 21

Ab¨vb¨ (wjLyb) [Other: specify].......................................................... 77

Rvwb bv[DK]................................................................................. 99

cÖ‡hvR¨ bq[Not applicable]…………………….................................. 88

- 1. me©‡kl gvwm‡Ki mgq gvwm‡Ki KvcowU cybivq e¨env‡ii Rb¨ †Kv_vq ïwK‡qwQ‡jb? **(GKvwaK DËi MÖnb‡hvM¨)** [For repeated use of menstrual cloth, where did you dry the menstrual cloth during last period? **(mark all that apply)**]

N‡ii wfZ‡i jywK‡q †i‡L[Inside the house but hiding somewhere]......... 1

N‡ii wfZ‡i Ges ‡Lvjv †gjv [Inside the house but open place]................. 2

N‡ii evwn‡i m~‡h©i Av‡jv‡Z[Outside the house and in sunlight]............... 3

N‡ii evwn‡i jywK‡q †i‡L[Outside the house but hiding somewhere]....... 4

ivbœv N‡ii Pzjvq [In side kitchen over cocking stove]...............................5

Uq‡j‡Ui wfZ‡i [Inside the toilet]........................................................ 6

†MvmjLvbvi wfZ‡i [Inside the bathroom]............................................... 7

e¨envh© Kvc‡oi wb‡P †i‡L [Underneath regular cloth]....................................8

Ab¨vb¨ (wbw`©ó K‡i wjLyb)[Other: specify]............................................... 77

Rvwb bv[DK]...................................................................................... 99

- 1. gvwm‡Ki mgq gvwm‡Ki KvcowU cybivq e¨env‡ii Rb¨ †Kv_vq ‡i‡L ïKvb? [For repeated use of menstrual cloth, generally where do you dry the menstrual cloth?]

| A | ï®‹ †gŠmy‡g [In dry season] |
| --- | --- |
| B | kxZ ‡gŠmy‡g[In winter] |
| C | elv© ‡gŠmy‡g[In rainy season] |

N‡ii wfZ‡i jywK‡q †i‡L[Inside the house but hiding somewhere]......... 1

N‡ii wfZ‡i Ges ‡Lvjv †gjv [Inside the house but open place]................. 2

N‡ii evwn‡i m~‡h©i Av‡jv‡Z[Outside the house and in sunlight]............... 3

N‡ii evwn‡i jywK‡q †i‡L[Outside the house but hiding somewhere]....... 4

ivbœv N‡ii Pzjvq [In side kitchen over cocking stove]...............................5

Uq‡j‡Ui wfZ‡i [Inside the toilet]........................................................ 6

†MvmjLvbvi wfZ‡i [Inside the bathroom]............................................... 7

e¨envh© Kvc‡oi wb‡P †i‡L [Underneath regular cloth]........................... 8

Ab¨vb¨ (wbw`©ó K‡i wjLyb)[Other: specify]............................................... 77

Rvwb bv[DK]...................................................................................... 99

cÖ‡hvR¨ bq[Not applicable]…………………….................................. 88

- 1. me©‡kl gvwm‡Ki mgq e¨eüZ KvcowU cieZx©‡Z e¨env‡ii Rb¨ Avcwb ‡Kv_vq ‡i‡L‡Qb? [Where didyou preserve this cloth for next use during your last period?].....................................

¯^vfvweKfv‡e Ab¨ Kvc‡oi gZ [Normally like other clothes].................. 1

jywK‡q[At a hidden place]................................................................. 2

weQvbvi wb‡P [Under bed]....................................................................... 3

j¨vwUª‡bi g‡a¨ [Inside the latrine]........................................................... 4

Ab¨vb¨ (wbw`©ó K‡i wjLyb)[Other: specify]................................................ 77

- 1. me©‡kl gvwm‡Ki mgq e¨eüZ KvcowU cieZx©‡Z e¨env‡ii Rb¨ Avcwb wK, cwjw_b/Kvco/KvMR w`‡q gywo‡q iv‡Lb? [During your last period, did you wrap this cloth by any plastic/polythenefor next use?]

bv [No]................................. 0

n¨uv[Yes]............................. 1

Rvwb bv[DK]........................... 99

- 1. ~~Dropped~~
  2. hw` Avcwb gvwm‡Ki Rb¨ e¨eüZ `ªe¨wU µq K‡i e¨envi K‡ib Zvn‡j Zvi Rb¨ cÖwZgv‡m Avcbvi KZ UvKv e¨q Ki‡Z nq? [If you need to buy any menstrual product then how much do you pay a month for using your menstrual products?]

[‡KvW: 88=cÖ‡hvR¨ bq (Code: 88=Not applicable)]

UvKv [Taka]....................................

Rvwbbv [Don’t know].................. 99

webvgy‡j¨ [Free of cost]........................ 00

cÖ‡hvR¨ bq [Not applicable]............. 88

*Skip Note:* ***hw` 230Gi DËi 88 nq, Z‡e 233G P‡j hvb***

*[If the answer of 230 is 88 then skip to 233]*

*Skip Note:* ***hw`230Gi DËi 00 nq, Z‡e 232G P‡j hvb***

*[If the answer of 230 is 00 then skip to 232]*

- 1. gvwm‡Ki Rb¨ e¨eüZ `ªe¨wU mvavibZ †K wK‡b _v‡Kb? [Who usually buys the menstrual products?]...............................................................................................

Avcwb wb‡R [Yourself].............................................................. 1

gv [Mother]............................................................................ 2

bvbx/`v`x [Grand-mother]........................................................ 3

evev [Father]............................................................................ 4

‡evb/fvex [Sister/Sister-in law]................................................ 5

fvB [Brother]........................................................................... 6

Ab¨ AvZ¥xq-gwnjv [Other relatives-female]................................ 7

Ab¨ AvZ¥xq-cyiæl [Other relatives-male].................................... 8

eÜz[Friend]............................................................................. 9

cÖwZ‡ekx [Neighbours].............................................................. 10

Ab¨vb¨ (wbw`©ó K‡i wjLyb)[Other (specify)].................................. 77

Rvwbbv [Don’t know].................. 99

- 1. gvwm‡Ki Rb¨ e¨eüZ `ªe¨wU mvavibZ †Kv_v †_‡K wK‡b/‡c‡q _v‡Kb? [Where does this person usually buy or obtain the product?]........................................................................

dv‡g©mx †_‡K [Local pharmacy]......................... 1

GjvKvi †`vKvb †_‡K [Local shop]...................... 2

mycvi kc †_‡K [Super shop]............................. 3

evRvi †_‡K [Market]....................................... 4

‡Kvb gwnjvi †_‡K [Other women]..................... 5

GbwRI/miKvi †_‡K [NGO/govt.]...................... 6

nmwcUvj/wK¬wbK †_‡K [Hospital or Clinic]........... 7

¯‹zj †_‡K [School]........................................... 8

Ab¨vb¨ (wbw`©ó K‡i wjLyb)[Other (specify)].......... 77

Rvwbbv [Don’t know]..................................... 99

- 1. Avcwb Rv‡bb †h gvwm‡Ki mgq i³cvZ †kvl‡Yi Rb¨ A‡bK ai‡bi m¨vwbUvwi cY¨ i‡q‡Q| me©‡kl gvwm‡Ki K_v wPšÍv K‡i Avgv‡`i GKUz ejyb Avcwb †h cY¨wU e¨envi K‡iwQ‡jb Zv‡Z Avcwb KZUv mš‘ó? (DËi ¸‡jv c‡o ‡kvbv‡Z n‡e) [As you know, there are multiple options for sanitary products that exist to absorb blood during menstruation. Think about your most recent period. How satisfied were you with the products you used?]

1= Amš‘ó [unsatisfied]

2=‡gvUvgywU Amš‘ó [somewhat unsatisfied]

3=Amš‘óI bv Avevi mš‘óI bv [indifferent]

4=‡gvUvgywU mš‘ó [somewhat satisfied]

5=mš‘ó [satisfied]

- 1. **gvwm‡Ki Rb¨ e¨envh© `ªe¨vw` m¤ú‡K© DËi`vZvi cQ›` [Preferred menstrual product by respondent]**


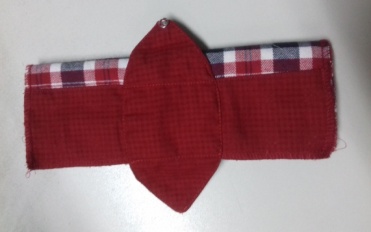

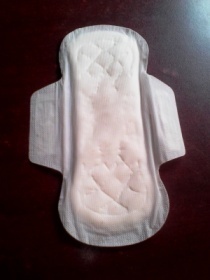

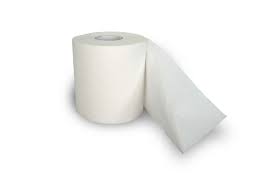


wUmy¨ †ccvi

m¨vwbUvix c¨vW

cybe©¨envi ‡hvM¨ c¨vW


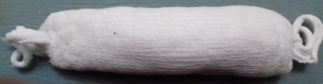

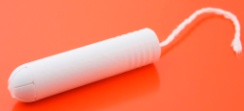

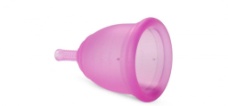


m¨vwbUvix c¨vW (PSTC)

gvwm‡Ki Rb¨ e¨eüZ Kvc

U¨v¤úyb


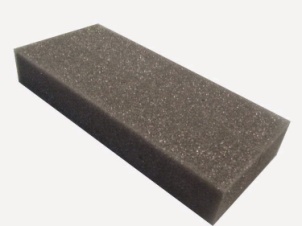

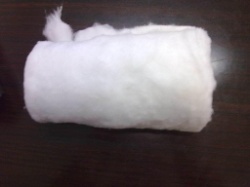


Zzjv

¯cÄ


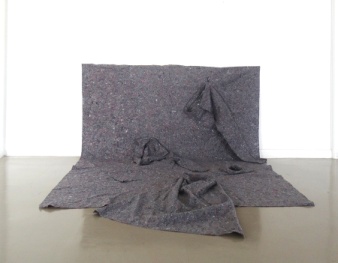

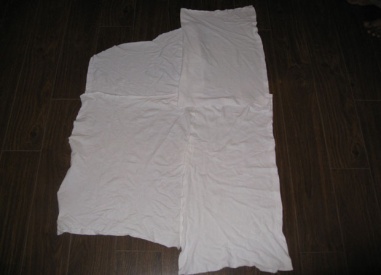


bZzb Kvco

Mv‡g©‡›Um Gi e¨eüZ Kvc‡oi UzKiv


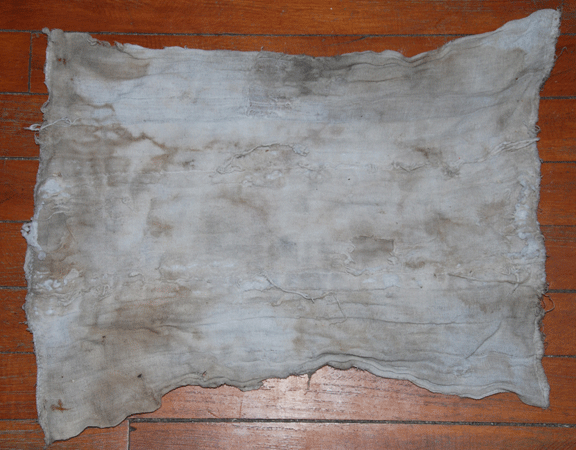


cyivZb Kvco

| Question | gvwmK PjvKvjxb mg‡q Avcwb wK ai‡bi `ªe¨ e¨envi Ki‡Z cQ›` Ki‡eb?[What types menstrual products will you prefer/comfort to use during menstruation] | bv [No]........... 0  n¨uv[Yes]......... 1 | ‡Kb Avcwb GUv e¨envi Ki‡Z cQ›` Ki‡eb? (GKvwaK DËi MÖnb‡hvM¨)[Why will you prefer to use this? (Multiple answer allowed here)] (** please follow below code) |
| --- | --- | --- | --- |
|  | cyivZb Kvco (Kvc‡oi UzKiv)[Old Cloth (rag)] |  |  |
|  | bZzbKvco[New cloth] |  |  |
|  | cybe©¨envi ‡hvM¨ c¨vW [Reusable Manufactured Sanitary Pads] |  |  |
|  | 1. m¨vwbUvix c¨vW (cybe©¨envi ‡hvM¨ bq)[Disposable Sanitary Pads] |  |  |
|  | 1. m¨vwbUvix c¨vW (PSTC) (cybe©¨envi ‡hvM¨ bq)[Disposable Sanitary Pads(PSTC)] |  |  |
|  | U¨v¤úyb [Tampons] |  |  |
|  | gvwm‡Ki Rb¨ e¨eüZ Kvc[Menstrual cup] |  |  |
|  | ¯cÄ[Sponges] |  |  |
|  | Zzjv [Cotton/wool] |  |  |
|  | wUmy¨ †ccvi [Tissue paper] |  |  |
|  | Mv‡g©‡›Um Gi e¨eüZ Kvc‡oi UzKiv [Jhute of garments] |  |  |
|  | ‡Kvb wKQzBbv [Nothing] |  |  |
|  | Ab¨vb¨ (wjLyb)[Other: Specify:] |  |  |

**** Use below code for 234**

Avivg`vqK [Comfortable]....................................... 1

m¯Ív [Low Cost]..................................................... 2

UvKv jv‡M bv[No need to pay]..................................... 3

memgq cvIqv hvq[Available].................................... 4

gv‡qi wmØvšÍ Abyhvqx [Decided by mother]................... 5

Pjv‡divq myweav [Easy to move].................................. 6

‡avqvi cÖ‡qvRb nq bv [No need to wash]....................... 7

‡avqv hvq [Washable]..................................................... 8
mn‡R †d‡j †`qv hvq [Easy to dispose]............................ 9
cwi‡ek evÜe [Environmentally Friendly]........................ 10
Qwo‡q/‡j‡M hvq bv [Stainproof]........................................................................ 11

‡kvlY ÿgZv fvj [Absorbent capacity good]...................................................... 12

RxevYyi msµgb Kg n‡e [Reduce germs infection]................................................ 13

‡hvbxc‡_i msµgb Kg n‡e[Reduce urinary tract infections].................................. 14

`yM©Ü Qov‡e bv[Will stop spreading bad smell].................................................... 15

AwaKZi †MvcbxqZv eRvq _v‡K [More privacy]..................................................... 16

mvgvwRK gh©v`v A¶zbœ _vK‡e [Social status]............................................................ 17

mn‡R ivLv Ges †d‡j †`qv hvq [Easy to put on and off].......................................18

Ab¨vb¨ (wjLyb)[Other: Specify:]....................................................................... 77

- 1. ~~Dropped~~
  2. ~~Dropped~~
  3. me©‡kl gvwmK PjvKvjxb mg‡q gvwm‡Ki Rb¨ Avcwb KZw`b ¯‹zj ev` w`‡q‡Qb? [How many days did you miss the school in the last menstrual period due to menstraution? (If answer is “00” then skip to 239)]............................................................

*Skip Note:* ***hw` 237Gi DËi 00 nq, Z‡e 238.a G P‡j hvb***

*[*If q237 answer is “00” then skip to ***238.a****]*

- 1. me©‡kl gvwm‡Ki mgq wK ai‡bi mgm¨vi Rb¨ ¯‹zj ev` w`‡Z n‡qwQj? **(GKvwaK DËi MÖnb‡hvM¨)** [What types of problem did you face regarding menstruation for missing school during last menstrual period? **(Multiple answers allowed here)**]............................................................................

gvwm‡K e¨eüZ `ªe¨vw` cvëv‡bvi RvqMv bvB [No place to change

the menstrual product].............................................................................. 1

c¨vW bvB [No available pad]....................................................................... 2

chv©ß cvwbbvB[No water available]....................................................... 3

evi/Zij mvevb bvB[No soap/liquid soap].............................................. 4

Avwg A¯^w¯Í†eva Kwi[I do not feel comfortable]........................................ 5

Avwg Amy¯’ _vwK [I remain sick]............................................................... 6

cÖPzi i³ÿiY nq [Excessive bleeding]..................................................... 7

hvZvqvZ mgm¨v [Unavailability of transport] ............................................. 8

Qwo‡q/‡j‡M hvIqvi f‡q _vwK [Afraid of visible menstrual leaks] ..................... 9

cvwievwiK wb‡lavÁv [Family barriers] ......................................................... 10

ag©xq wb‡lavÁv [Religious barriers] ........................................................... 11

wkÿK evwo‡ZB _vK‡Z e‡j‡Q [My teacher told me to stay at home]................ 12

Ab¨vb¨(wbw`©ó Ki‡Z n‡e) [Other (specify)].................................................... 77

238.a me©‡kl gvwm‡Ki mgq, ¯‹z‡j ¯^vfvweK K¬vm PjvKvjxb Ae¯’vq (cixÿvi mgq Qvov) gvwm‡Ki Kvi‡b †Zvgv‡K KZ¸‡jv K¬vm ev` w`‡Z n‡qwQj (hw` DËi`vZv ‡Kvb K¬vm ev` bv w`‡q _v‡K Zvn‡j “88” †KvW Kiæb)? [During your last menstrual period, how many regular classes (except exam) did you miss due to menstruation (If the respondebt did not miss any class then put code “88”)?].............

- 1. MZ wZb gv‡mi g‡a¨ Avcbvi †Kvb mncvVx gvwm‡Ki Rb¨ ¯‹zj/K¬vm ev` w`‡q‡Qb wK? [Did any of your fellow friends miss the school/class in the last 3 months due to menstruation?].........................................................

bv[No].................................. 0

n¨uv[Yes]............................... 1

Rvwbbv [DK]………...........…. 99

- 1. me©‡kl gvwm‡Ki mgq, Avcbvi wK e¨v_v n‡qwQj? [During your most recent period, did you experience pain during menstruation?] ............

bv[No].................................. 0

n¨uv[Yes]............................... 1

hw` DËi 0 nq Zvn‡j cieZx© †mKkv‡b P‡j hvb [If answer is “0” Skip to next section]

- 1. hw` n¨vu nq Zvn‡j GUv KZUv Zxeª wQj? [If yes, then ask them about the intensity of their pain]

g„`y [Mild]...................................................... 1

‡gvUvgywU mn¨ Kivi gZ [Moderate]..................... 2

Zxeª [Severe]................................................... 3

Ab¨vb¨ (wbw`©ó K‡i wjLyb)[Other (specify)].......... 77

Rvwbbv [Don’t know]..................................... 99

- 1. hw` n¨vu nq, Zvn‡j KZ w`b a‡i e¨v_v wQj? [If yes, how long the pain lasted?]

GKw`b [One day]............................................. 1

`yBw`b [Two days]..................... 2

wZbw`b [Three days]................................................... 3

Ab¨vb¨ (wbw`©ó K‡i wjLyb)[Other (specify)].......... 77

Rvwbbv [Don’t know]..................................... 99

**[Section 4:** gvwmK PjvKvjxb wewawb‡la Ges ¯‹z‡ji my‡hvM myweav **[Forbidden activities and school facilities]]**

| Ques no. | Questions | Answer:  n¨uv[Yes]............1  bv[No]............. 2 |
| --- | --- | --- |
| - 1. gvwmK PjvKvjxb mg‡q †Kvb †Kvb KvR¸‡jv Ki‡Z wb‡la Kiv nq?[What are the activities forbidden for you during menstruation ? | | |
|  | wbw`©ó wKQz ¯’v‡b †h‡Z cv‡i bv [Not go to certain places] |  |
|  | wbw`©ó wKQz wRwbm ai‡Z cv‡i bv (weQvbvq ï‡Z/ai‡Z †`q bv) [Not touch certain things (i.e don’t get up bed) ] |  |
|  | wbw`©ó wKQz Lv`¨ MÖnY Ki‡Z cv‡i bv [Not eat certain foods] |  |
|  | ivbœv Ki‡Z †`qv nq bv[Not allowed to cook] |  |
|  | evwn‡i †h‡Z †`qv nq bv [Not allowed to go out] |  |
|  | ¯‹z‡j †h‡Z †`qv nq bv [Not allowed to go to school] |  |
|  | agx©q KvR ‡_‡K weiZ _vKv [Not allowed to religious activities] |  |
|  | gvwm‡Ki e¨eüZ Kvco/`ªe¨ I AvÛviIqvi evwn‡i ïKv‡Z †`qv wb‡la[Not allowed to dry menstrual rags and underwear in open place] |  |
|  | gvwm‡Ki mgq †Q‡j‡`i mv‡_ K_v ejv wb‡la [Not allowed to talk to boys/males] |  |
|  | gvwm‡Ki mgq AÜKvi ¯’v‡b hvIqv wb‡la [Not allowed to go in dark] |  |
|  | wKQzB wbwl× bv[Nothing] |  |
|  | fvix KvR Kiv wb‡la [Not allowed to do heavy work] |  |
|  | ‡Ljv-a~jv Kiv wb‡la [Not allowed to play] |  |
| 77. | Ab¨vb¨ (wbw`©ó K‡i wjLyb)[Others specify] |  |

- 1. me©‡kl gvwm‡Ki mgq gvwm‡Ki `vM wK Avcbvi Kvc‡o †j‡MwQj? [Did you experience any leakage or staining in your outer garments during their last menstruation?]...................................

bv[No].................................. 0

n¨uv[Yes]............................... 1

Rvwbbv [DK]…………............ 99

- 1. Avcwb wK g‡b K‡ib ¯‹z‡j †h me my‡hvM myweav Av‡Q Zv gvwmK msµvšÍ cwi®‹vi-cwi”QbœZv e¨e¯’vcbv/iÿvi Rb¨ chv©ß ev h‡_ó?[Do you think that the school facilities are appropriate for managing menstrual hygiene?]............................................................................................................

FRA Gi Rb¨ ‡bvU: DËi ¸‡jv c‡o †kvbvb Ges †`Lvb| [Note for FRA: Please show and read the answer]

1= LyeB Achv©ß [very inadequate]

2=‡gvUvgywU Achv©ß [somewhat inadequate]

3=Achv©ßI bv Aevi chv©ßI bv [neither adequate nor inadequate]

4=‡gvUvgywU chv©ß [somewhat adequate]

5=chv©ß [adequate]

- 1. gvwmK ¯^v¯’¨ e¨e¯’vcbvi Rb¨ Avcbvi ¯‹z‡j †Kvb c„_K GW‡j‡m›U/ eqtmwÜ Kb©vi Av‡Q wK? [Is there any separate adolescent corner in your school for menstrual hygiene management?]

bv[No].................................. 0

n¨uv[Yes]............................... 1

Rvwbbv [DK]…………............ 99

hw` DËi 0/99 nq Zvn‡j P‡j q408 G hvb

[If answer is 0/99 then skip to q408]

- 1. †K GUv †`Lv‡kvbv K‡ib? (GKvwaK DËi MÖnb‡hvM¨) [Who maintain this? (Multiple answers allowed here)]..................

†RwbUi/Avqv [Jenitor/aya]............................................. 1

wkwÿKv [Teacher]...................................................... 2

QvÎxiv [Students]…………....................................... 3

Rvwbbv [DK]…………............................................. 99

Ab¨vb¨ (wbw`©ó K‡i wjLyb) [Others (Specify)] ................. 777

- 1. GLb ch©šÍ Avcwb wK ai‡bi gvwm‡Ki `ªe¨ ¯‹zj †_‡K †c‡q‡Qb? [What type of MHM product did you get from your school till now?]

| Sl. | aiY [Type ]  FRA Gi Rb¨ ‡bvU: DËi ¸‡jv c‡o †kvbvb\| [Note for FRA: Please read the answer] | msL¨v[Number] | Avcwb wK G¸‡jv eZ©gv‡b e¨envi K‡ib? [Do you use currently these products?]  bv[No]... 0  n¨uv[Yes]........... 1  hw` DËi 0 nq Zvn‡j P‡j q409 G hvb [If answer is 0 then skip to q409] | hw` bv nq Zvn‡j, †Kb e¨envi K‡ib bv? **(GKvwaK DËi MÖnb‡hvM¨)** [If no, then why not? **(Multiple answers allowed here)** ]  * wb‡Pi †`qv †KvW¸‡jv e¨envi Kiæb [please use below code] |
| --- | --- | --- | --- | --- |
|  | DBs c¨vW [Wing Pads] |  |  |  |
|  | c¨vw›U [Underwear] |  |  |  |
|  | cybe©¨envi ‡hvM¨ Kvc‡oi c¨vW [Re-usable cloth pad] |  |  |  |
|  | bxj cøvw÷K e¨vM (MHM c¨v‡KU) [Blue plastic bag (MHM pack)] |  |  |  |
|  | g¨v‡R›Uv e¨vM [Magenta bag] |  |  |  |
|  | Menstrual Calender |  |  |  |
|  | Ab¨vb¨ (wbw`©ó K‡i wjLyb) [Others (Specify)] |  |  |  |

*** wb‡Pi †`qv †KvW¸‡jv Kjvg-5 Gi Rb¨ e¨envi Kiæb [Please use following code for column -5]

Avivg`vqK bv [Not comfortable]...................................................... 1

Avwg G¸‡jv cQ›` Kwi bv [Don’t like these product] ....................... 2

Avwg Ab¨ m¨vwbUvix c¨vW e¨envi Kwi [I use other sanitary product] ............ 3

gv e¨envi Ki‡Z †`q bv [Mothers not allow using] ............................... 4

ay‡Z nq [Need to wash] ..................................................................... 5

e¨envi Kiv KwVb [Difficult to use] ....................................................... 6

‡bqvi ci GLbI gvwmK nqwb [Not yet menstruation after getting the pad] ........ 7

Ab¨ KvD‡K w`‡q w`‡qwQ [Give to someone] ........................................... 8

Ab¨vb¨ (wbw`©ó K‡i wjLyb) [Others (Specify)]......................................... 777

- 1. Avcbvi ¯‹z‡j gvwm‡Ki e¨eüZ `ªe¨ †djvi Rb¨ ‡Kvb cvBc wWm‡cvmvj wm‡÷g Av‡Q wK? [Have you heard about a piped disposal system in your school?].....................................................

bv [No].................................. 0

n¨uv [Yes]............................... 1

Rvwbbv [DK]…………............ 99

hw` DËi nq 0/99 Zvn‡j 412 P‡j hvb

[If answer is 0/99 then skip to 412]

- 1. Avcwb wK GUv KLbI e¨envi K‡i‡Qb? [Have ever used the disposal system?]].................

bv [No].................................. 0

n¨uv [Yes]............................... 1

Rvwbbv [DK]…………............ 99

hw` DËi 1 nq Zvn‡j q412 P‡j hvb

[If answer is 1 then skip to 412]

- 1. hw` bv nq Zvn‡j, †Kb e¨envi K‡ib bv? (**GKvwaK DËi MÖnb‡hvM¨**) [If no, then why not? **(Multiple answers allowed here)**] ........................................

¯‹z‡j cwieZ©b Kwi bv [Don’t change menstrual material while at school]... 1

Avwg evwo‡Z wM‡q cwieZ©b Kwi [I change my pad after returning home] ... 2

Avwg cybe©¨envi ‡hvM¨ `ªe¨ e¨envi Kwi [I use reusable menstrual materials] ... 3

cvBc wWm‡cvmvj wm‡÷‡g mgm¨v wQj [There was a problem with the piped disposal system] .4

Avwg cvBc wWm‡cvmvj wm‡÷g m¤ú‡K© Rvwbbv [I don’t kow what the piped disposal system] 5

GUvi mwVK e¨envi Avgvi Rvbv wQj bv [I was unsure of how to use it properly] ............... 6

Avwg e¨eüZ c¨vWwU †h †Kvb ¯’v‡b †d‡j w`B [I threw the used pad elsewhere] ............... 7

Ab¨vb¨ (wbw`©ó K‡i wjLyb) [Other (specify):]........................................................... 777

- 1. Avcbvi ¯‹z‡j QvÎ-QvÎx‡`i Rb¨ †Kvb cÖ‡kœvËi e· Av‡Q wK? [Is there any question-answer box available for the students?] ...........................................................................................

bv[No].................................. 0

n¨uv[Yes]............................... 1

Rvwbbv [DK]…………............ 99

hw` DËi 0/99 nq Zvn‡j q415 †Z P‡j hvb

If answer is 0/99 then skip to 415

- 1. Avcwb wK KLbI †mLv‡b †Kvb cÖkœ wj‡L‡Qb? [Have you ever written a question there?] ......

bv[No].................................. 0

n¨uv[Yes]............................... 1

Rvwbbv [DK]…………............ 99

hw` DËi 0/99 nq Zvn‡j q415 P‡j hvb

If answer is 0/99 then skip to 415]

- 1. hw` n¨vu nq, Avcwb wK h_vh_ DËi †c‡qwQ‡jb? [If yes, did you get your appropriate answer?]

bv[No].................................. 0

n¨uv[Yes]............................... 1

Rvwbbv [DK]…………............ 99

- 1. Avcwb wK eq:mwÜKvjxb †Kvb eB ¯‹zj †_‡K †c‡q‡Qb? [Did you receive puberty book from your school?] ..........................................................................................................................

bv[No].................................. 0

n¨uv[Yes]............................... 1

Rvwbbv [DK]…………............ 99

hw` DËi 1 nq Zvn‡j q417 †Z P‡j hvb

[If answer is 1 then skip to 417]

- 1. eq:mwÜKvjxb GB eBwU wK c‡owQ‡jb? [Did you read the puberty book?] .........................

bv[No].................................. 0

n¨uv[Yes]............................... 1

Rvwbbv [DK]…………............ 99

- 1. Avcwb wK cÖwZw`b ¯‹z‡j Avmvi ci Qvc †`qvi hš¿wU‡Z Avcbvi Av½y‡ji Qvc †`b? [Did you scan your finger regularly after coming at school?] ...........................................................

bv[No].................................. 0

n¨uv[Yes]............................... 1

Rvwbbv [DK]…………............ 99

hw` DËi 0/99 nq Zvn‡j cieZx© †mKkv‡b P‡j hvb

[If answer is 0/99 then skip to next section]

- 1. hw` DËi bv nq Zvn‡j, †Kb †`b bv? [If answer is No, why is the reason?]

ZvwjKv fy³ bv [Not registered].......................................... 1

Qvc w`‡Z fz‡j hvB [Forgot to scan]................................... 2

j¤^v jvBb [Long queue]............................................................ 3

hš¿wU‡Z we`yr ms‡hvM wQj bv [Device had no connection].................4

Qvc wb‡Z Amdj [Unsuccesful scanning]................................. 5

Ab¨vb¨ (wbw`©ó K‡i wjLyb) [Others (Specify)] ............................. 777

**†mKkb 5 gvwmK msµvšÍ Z_¨ I Z‡_¨i Drm mg~n**

**[Section 5: Information and sources of information regarding menstruation]**

- 1. ~~Dropped~~
  2. Avcwb †Kv_v †_‡K gvwmK m¤ú‡K© Z_¨ †c‡qwQ‡jb (GKvwaK DËi n‡Z cv‡i)? [Where did you get the information about menstruation? (Multiple answers allowed here)] ..............

gv[Mother] ............................................................. 1

evev[Father] ............................................................ 2

`v`x/bvbx [Grandmother] ............................................ 3

eÜz [Friend] .............................................................. 4

PvPx/gvgx/Lvjv [Aunty]................................................ 5

wkÿK [Teachers] ...................................................... 6

‡evb/ fvex [Sister/ Sister-in law]................................ 7

Wv³vi/bvm© [Doctor/Nurse]......................................... 8

wUwf/‡iwWI [TV/Radio] ............................................. 9

cvV¨eB †_‡K [Reading/curriculum]........................... 10

cvV¨ eB‡qi evB‡i [Extra-curricular].............................. 11

wgwWqv †_‡K [Media].................................................. 12

B›Uvi‡bU †_‡K[Internet]................................................ 13

cb©MÖvwdK g¨vMvwRb †_‡K[Pornographic Magazine].......... 14

mncvVx‡`i †_‡K [Group/peer education in school].......15

cÖwZ‡ekx[Neighborhood]...........................................16

¯^v¯’¨Kg©x [Health worker]...........................................17

¯^v¯’¨‡K›`ª [Health care centre].....................................18

eq:mwÜKvjxb eB †_‡K [From puberty book].............................. 19

AvBwmwWwWAviwei †mkb†_‡K [Session of icddrb]..................20

gvwmK m¤ú‡K© Rvwbbv [Don’t know about menstruation]..................21

Ab¨vb¨ (wbw`©ó K‡i wjLyb)[Other (Specify):]...................... 77

- 1. Avcwb gvwmK m¤ú‡K© wK ai‡Yi Z_¨ ‡c‡qwQ‡jb? (GKvwaK DËi n‡Z cv‡i) [What types of information did you receive about menstruation?(Multiple answers allowed here)]......................

GUv bvix‡`i GKwU ¯^vfvweK welq GUv wb‡q fq bv †c‡Z [Don’t worries about menstruation

it’s a normal phenomenon of a female] ................................................. 1

G mgq kvwiwiK †h †Kvb ai‡Yi mgm¨v n‡Z cv‡i †mB m¤ú‡K© [May occur any physical complication].......................................................................................... 2

‡hvbx c_ w`‡q i³cvZ nq [Blood exits via the vagina]............................................ 3

mvgvwRK ev agx©q wewa-wb‡la m¤ú‡K© [Social or religious norms] ...................... 4

GB mg‡q wKfv‡e cwi®‹vi-cwi”QbœZv eRvq ivL‡Z n‡e †mB m¤ú‡K© [How to maintain hygiene during menstruation]....................................................................................... 5

wK ai‡Yi c¨vW/Kvco e¨envi Ki‡Z n‡e †mB m¤ú‡K© [What types of materials should be use during menstruation] ....................................................................................... 6

wKfv‡e c¨vW/Kvco e¨envi Ki‡Z n‡e [How to use cloth/pad during menstruation]... 7

wKfv‡e Ges †Kv_vq e¨eüZ c¨vW/Kvco ‡dj‡Z n‡e [How and where to dispose the used cloth/pad during menstruation] ........................................................................ 8

‡Kvb ai‡bi Z_¨ cvBwb [Didn’t get any information]............................................. 9

mylg Lvevi ‡L‡Z [To eat balanced food] ..................................................... 10

gvwm‡Ki Kvi‡b †Kvb mgm¨v n‡j Wv³v‡ii civgk© wb‡Z [Getting any trouble due to menstruation, then consult with the docto] .......................................................................... 11

Ab¨vb¨ (wbw`©ó K‡i wjLyb)[Other (Specify):] .......................................................... 77

503.b. gvwmK ïiæi Av‡M gvwmK msµvšÍ wel‡q Avcwb †Kvb wKQz Rvb‡Zb wK?(GgbwK hw` Avcbvi GLbI gvwmK ïiæ bv n‡q _v‡K)[Did you know about menstruation before you started menstruating (even if you not started menstruation?].......................................................................................

bv[No].................................. 0

n¨uv[Yes]............................... 1

GLb Avwg Avcbv‡K eq:mwÜ, cÖ_g ev Av`¨gvwmK Ges gvwmK m¤ú©wKZ wel‡q wKQ zcÖkœ wRÁvmv Ki‡Z PvB [***Now I will ask you some questions regarding puberty, menarche and menstruation.]***

FRA Gi Rb¨ ‡bvU: wKD KvW©wU †`Lvb | [Note for FRA: Please show the que card]

| - 1. Avcwb eq:mwÜ, cÖ_g ev Av`¨gvwmK Ges gvwmK m¤ú©wKZ wel‡q K_v ej‡Z Kvi mv‡_ KZUv K_v ej‡Z ¯^v”Q›`¨‡eva K‡ib? [How much do you comfortable with whom to discuss regarding **puberty, menarche, and menstruation**?] | | | | | | |
| --- | --- | --- | --- | --- | --- | --- |
|  | Item | Lye A¯^w¯Í‡eva Kwi [Very Uncomfortable]=1 | A¯^w¯Í‡eva Kwi [Uncomfortable]  =2 | ¯^w¯Í I †eva Kwibv Avevi A¯^w¯Í I †eva Kwibv [Neither or  Not sure]=3 | ¯^v”Q›`¨‡eva Kwi [Comfortable]  =4 | Lye ¯^v”Q›`¨‡eva Kwi [Very  Comfortable]=5 |
| 504.1. | ¯‹z‡ji wkÿK [Teacher at school] |  |  |  |  |  |
| 504.2. | Wv³vi/MvB‡bvKjwR÷ [Doctor/gynaecologist] |  |  |  |  |  |
| 504.3. | bvm© [Nurse] |  |  |  |  |  |
| 504.4. | ¯‹zj K¬ve [School clubs] |  |  |  |  |  |
| 504.5. | evÜex [Friend] |  |  |  |  |  |
| 504.6. | gv [Mother] |  |  |  |  |  |
| 504.7. | evev [Father] |  |  |  |  |  |
| 504.8. | fvB [Brother] |  |  |  |  |  |
| 504.9. | ‡evb/fvex [Sister/ Sister-in law] |  |  |  |  |  |
| 504.10. | PvPx/gvgx/dzcy/Lvjv (Avcb) [Aunt] |  |  |  |  |  |
| 504.11. | PvPv/gvgv/dzcv/Lvjy (Avcb) [Uncle] |  |  |  |  |  |
| 504.12. | `v`x/bvbx [Grandmother] |  |  |  |  |  |
| 504.13. | `v`v/bvbv [Grandfather] |  |  |  |  |  |
| 504.77 | Ab¨vb¨ (wbw`©ó K‡i wjLyb)[Other (Specify)] |  |  |  |  |  |

wb¤œwjwLZ **Dw³ ¸‡jvi mv‡_ Avcbvi gZvg‡Zi gvbUv Avgv‡`i GKUz e‡jb|** **Avcwb wK GB Dw³ ¸‡jvi mv‡_ KZUv GKgZ ev wØgZ |[Please tell us your level of agreement with the following statement. Do you agree, disagree, somewhat agree or somewhat disagree?]**

FRA Gi Rb¨ ‡bvU: wKD KvW©wU †`Lvb | [Note for FRA: Please show the que card]

wb¤œwjwLZ Q505-Q523 cÖ‡kœi Rb¨ **GB DËi¸‡jv e¨envi Kiæb** [Please use the below response options as card/flash card for Q505-Q523]

1 = LyeB GKgZ [strongly agree]

2 = GKgZ [agree]

3 = †gvUvgywU GKgZ [somewhat agree]

4 = †gvUvgywU wØgZ [somewhat disagree]

5 = wØgZ [disagree]

6 = LyeB wØgZ [strongly disagree]

- 1. .a. me©‡kl gvwm‡Ki mg‡q, Avcwb fq †c‡qwQ‡jb ‡h ¯‹z‡j gvwm‡Ki Kvi‡b Avcwb DË¨³/e¨½/nvwm Zvgvkvi Kvib n‡Z cv‡ib|[During your last period, you felt fear that you might get teased because of period at school.]

505.b. †g‡q‡`i‡K gvwmK wb‡q DË¨³/e¨½/nvwm Zvgvkv/VvÆv/we`ªæc Kiv Avcbv‡`i ¯‹z‡ji QvÎ-QvÎx‡`i Rb¨ LyeB ¯^vfvweK e¨vcvi| [It is common in your school for students to tease girls about menstruation]

- 1. me©‡kl gvwm‡Ki mg‡q, ¯‹z‡j gvwm‡Ki Kvi‡b Avcwb welbœZv/gbgiv fve Abyfe K‡iwQ‡jb [During your last period, you felt anxious at school because of your period]:
  2. me©‡kl gvwm‡Ki mg‡q, Avcwb ¯‹z‡j ¯^w¯Í‡eva K‡iwQ‡jb| [During your most recent period, you felt comfortable at school]
  3. me©‡kl gvwm‡Ki mg‡q, gvwm‡Ki Rb¨ K¬v‡k gb‡hvM w`‡Z Avcbvi mgm¨v n‡qwQj| [ During your most recent periods, you have felt distracted or had trouble concentrating in class due to period.]
  4. me©‡kl gvwm‡Ki mg‡q, gvwm‡Ki Kvi‡b K¬v‡ki wewfbœ Kh©µ‡g (wb‡R †_‡K `vuwo‡q DËi w`‡Z ev †ev‡W© wM‡q wjL‡Z) AskMÖnb Ki‡Z Avcbv‡K evavui m¤§yLxb n‡Z n‡qwQj| [During your most recent periods, my period has affected my participation in class—(For example: willingness to stand to answer questions or walk to the board, etc.)]
  5. ¯^vfvweK ˆ`bw›`b KvRKg© Ki‡Z ‡g‡q‡`i Rb¨ gvwmK †Kvb cÖwZeÜKZv/evuav bq| [Menstruation does not have to restrict a girl’s involvement in her normal daily activities]

| SL | Item |
| --- | --- |
|  | ~~Dropped~~ |
|  | gvwm‡Ki mgq Avwg evwo‡Z _vK‡ZB †ewk cQ›` Kwi [I prefer staying at home during my period] |
|  | gvwm‡Ki mgq Avwg Ab¨vb¨ w`‡bi †_‡K wb‡Ri Dci Kg AvZ¥wek¦vmx _vwK [During my period I feel less self-confident than other days] |
|  | gvwm‡Ki mgq Avwg kvwiwiK e¨qvg/ Ab¨vb¨ Kvh©µg Gwo‡q Pwj [During my period I avoid physical activity] |
|  | Avwg Avgvi cieZx© gvwmK wb‡q f‡q _vwK [I am afraid of my next period] |
|  | Avwg Avgvi gvwmK wb‡q Av‡iv †ewk Rvb‡Z PvB [I wish I would know more about my period] |
|  | gvwmK ‡g‡q‡`i e¨vcvi GUv wb‡q †Q‡j‡`i Av‡jvPbv Kiv DwPZ bq [Menstruation is a girls/woman’s matter that men or boys should not discuss] |
|  | gvwm‡Ki mgq †g‡qiv Acwi®‹vi/AïwP/bvcvK _v‡K [Girls are unclean/polluted during their menstruation] |
|  | gvwmK †g‡q‡`i my¯’Zvi jÿb/‡g‡q‡`i Rb¨ fvj [Menstruation is a healthy thing for a girl ] |
|  | gvwmK Avjøvni/m„wóKZv©i Awfkvc [Menstruation is a curse of God] |
|  | ‡g‡q‡`i ¯^v¯’¨m¤§Z gvwmK e¨e¯’vcbvi Rb¨ cÖwZgv‡m m¨vwbUvix cb¨ †Kbvi Rb¨ GKwU wbw`ó© cwigvb UvKv eivÏ _vKv DwPZ [It is reasonable for a family to spend money each month to purchase sanitary products for the girls/women to manage their menstrual hygiene ] |
|  | Avwg Rvwbbv eqmwÜKvjxb m¤ú‡K© mwVK Z_¨ Rbvi Rb¨ Kvi Kv‡Q †h‡Z n‡e [I feel I do not know whom to go to get accurate information about puberty ] |
|  | eqmwÜKvjxb mg‡q †h ai‡bi cwieZ©b ¸‡jv nq Avwg Zvi Rb¨ AcÖ¯‘Z [I feel unprepared for the changes that happen during puberty] |

- 1. hw` Avcbvi K¬v‡ki cvV¨ mnvwqKvq gvwmK msµvšÍ Z_¨ _v‡K Zvn‡j, ‡K GB K¬v‡m covb?[If your text book have any information regarding menstruation, who teaches these classes?] .............................................................

cyiæl wkÿK [Male teacher]......................................... 1

gwnjv wkÿK [Female teachers]................................... 2

Dc‡ii K¬v‡mi QvÎxiv [Senior female students] .............. 3

Dc‡ii K¬v‡mi QvÎiv [Senior male students] ................. 4

cvV¨ mnvwqKvqgvwmK msµvšÍ ‡Kvb Z_¨ †bB

[No information in the text book]..............................5

cov‡bv nq bv[Dont teaches].....................................6

Avwg Rvwbbv[I don’t know].......................................99

Ab¨vb¨ (wbw`©ó K‡i wjLyb) [Others specify].................... 77

- 1. hw` 524 Gi DËi 1/4nq Zvn‡j, QvÎ/cyiæl wkÿ‡Ki KvQ †_‡K G ai‡Yi welq m¤ú‡K© wkÿv ‡c‡Z Avcbvi †Kgb jv‡M? [If answer of 524is 1/4 then, how do you feel learning such issues from male teachers/male students?] .....................................................................................

Avwg A¯^w¯Í†eva Kwi[Feeling uncomfortable]............... 1

j¾v jv‡M [Feeling shy].......................................... 2

Lvivc jv‡M [Feeling bed]......................................... 3

fvj jv‡M [Feeling good].......................................... 4

‡Kvb cv_K¨© bvB [No difference]................................ 5

Ab¨vb¨ (wbw`©ó K‡i wjLyb) [Others specify].................... 77

- 1. ~~Dropped~~

Avcbvi ¯‹z‡j gvwmK wb‡q wK ai‡Yi cvV`v‡bi /wkÿv cÖ`v‡bi e¨e¯’v Av‡Q?(GKvwaK DËi n‡Z cv‡i) [What menstruation education sessions are provided for girls? (Multiple answers allowed here)] …....................................................................................................……

QvÎx‡`i Rb¨ Dc‡`k ev civgk© cÖ`vb [Counseling for girls]…………………… 1

QvÎx‡`i Rb¨ Avjv`v K¬v†ki e¨e¯’v [Separate class are arranged for girls]……… 2

wbqwgZK¬v†kie¨e¯’v [With regular classes]………………… ………………. 3

GbwRI/¯^v¯’¨Kgx©iv QvÎx‡`i Rb¨ Awa‡ek‡bi Av‡qvRb K‡ib [NGO/health

workers arrange sessions for girls]…………..……..…………………...… 4

‡RÛvi K¬ve Av‡jvPbv [Gender club discussion]………………………………... 5

wKQzB bv [Nothing]……… ………………………………………………… 6

fywgKv cvjb Kivi gva¨‡g [Role play]………… ………………………………. 7

B†Rj÷¨vÛ/ B†Rj ‡evW©/ wd¬cPvU© [Use easel stand/board or flip chart]…….. 8

cvIqvi c‡q›U Gi gva¨‡g [Use PPT slide show]…… ……….…… 9

Ab¨vb¨ (wbw`©ó K‡i wjLyb)[Others (specify)]………………………………… 77

cÖ‡hvR¨ bq[Not applicable]……….......................................................... 88

527.a. gvwmK wkÿv †mkb Gi welqe¯‘mg~n wK wQj? (GKvwaK DËi n‡Z cv‡i) [What were the content of the menstrual hygiene education session? ((Multiple answers allowed here)]

QvÎ-QvÎx‡`i g‡a¨ e¨w³MZ cwi¯‹vi-cwi”QbœZv †g‡b Pjvi ¸iæZ¡ welqK Av‡jvPbv

[Promoting importance of personal hygiene management among students] …....… 1

wcZvgvZvi fzwgKv welqK [Parental role] …......................................................… 2

¯‹z‡j Avmvi ci AvKw¯§K gvwmK n‡q †M‡j mnvqZv cvIqv welqK

[Supporting in managing sudden menstruation]...............................................… 3

Riæix cÖ‡qvR‡b gvwm‡Ki DcKib e¨env‡ii wbqš¿b welqK

[Monitoring the use of emergency products]....................................................… 4

eq:mwÜKvjxb cwieZ©b ¸‡jv wb‡q [Changes that happen during puberty].................................................................................................................... 5

Ab¨vb¨ (wbw`©ó K‡i D‡jøL Kiæb) [Other: specify] …........................................… 777

527.b. Avcbv‡`i ¯‹z‡j †Kvb ‡RÛvi K¬ve/KwgwU MVb Kiv n‡q‡Q wK ? [Do your school have any gender committee/club?]

bv[No].................................. 0

n¨uv[Yes]............................... 1

Rvwbbv [DK]…………............ 99

hw` DËi 0/99 nq Zvn‡j q528 P‡j hvb [If answer is 0/99 then skip to q528]

527.c. Kviv Kviv GB K¬ve/KwgwUi m`m¨?(GKvwaK DËi n‡Z cv‡i) [Who were the members in the gender committee/club? ( Multiple responses are allowed here)]

cÖavb wkÿK [Head Master] ….................................…………………. 1

mnKvix cÖavb wkÿK [Assistant Head Master]… ………….............…….. 2

¯‹zj e¨e¯’vcbv KwgwUi (Gm.Gg.wm) m`m¨ (mwPe) [SMC Member (Secretary…. . 3

¯‹zj e¨e¯’vcbv KwgwUi (Gm.Gg.wm) m`m¨ (mnKvix mwPe) [SMC Member (Assistant Secretary)]……………….................................................……. . 4

AwffveK m`m¨ (mfvcwZ) [PTA Chair]…………………… ………. 5

AwffveK m`m¨ (mnmfvcwZ) [PTA Co-Chair]………........... ………….. 6

wkÿK (AvPib cwieZ©‡bi †hvMv‡hvM welqK cÖwkÿYcÖvß) [Teacher (BCC Trained)].7

wkÿK (Ab¨vb¨) [Teacher (Other)]……… ………...............…………… . 8

Avqv/ZË¡veavqK [Janitor]………………….........................………. 9

¯‹zj †Kwe‡bU m`m¨e„›` [Cabinet Members]……………........... …………. 10

Ab¨vb¨ (wbw`©ó K‡i D‡jøL Kiæb) [Others (specify)....................................... 777Rvwbbv [DK]…………............ 99

527.d. ‡RÛvi K¬ve/KwgwU‡Z wK wel‡q Av‡jvPbv Kiv n‡q _v‡K? (GKvwaK DËi n‡Z cv‡i) [What are the topics discussed in gender committee meeting?( Multiple responses are allowed here)]

cÖKí ev¯Íevqb Ges Kvh©µgmgyn [Intervention and its activity] …....................… 1

cÖwZgv‡m wgwUs Av‡qvR‡bi mgqm~Px Ges e¨e¯’vcbv welqK [Schedule and arrangement of monthly meetings] ….......................................................................… 2

Abycw¯’wZ cwigv‡ci c×wZ/hš¿ iÿbv‡eÿY [Maintenance of absenteeism tracking system] …....................................................................................................… 3

cvBc wWmcRvj c×wZi iÿbv‡eÿb [Maintenance of piped disposal system] …… 4

†iwR÷vi LvZv wVKg‡Zv iÿbv‡eÿb Kiv nq wKbv Zvi ZË¡veavqb [Supervision of maintenance of register book] …...........................................................................… 5

gvwmK welqK me©‡kl †mkb Gi Ask wn‡m‡e cwiKíbv cÖ¯‘ZKiY [Preparation of the skits as part of the final session on menstruation] ….......................................… 6

QvÎ-QvÎx‡`i g‡a¨ e¨w³MZ cwi¯‹vi-cwi”QbœZv †g‡b Pjvi ¸iæZ¡ welqK Av‡jvPbv [Promoting importance of personal hygiene management among students] …..........… 7

wcZvgvZvi fzwgKv welqK [Parental role].................................................… 8

¯‹z‡j Avmvi ci AvKw¯§K gvwmK n‡q †M‡j mnvqZv cvIqv welqK [Supporting in managing sudden menstruation] …....................................................................… 9

Riæix cÖ‡qvR‡b gvwm‡Ki DcKib e¨env‡ii wbqš¿b welqK [Monitoring the use of emergency products] …...................................................................................… 10

cÖRbb Zš¿ Ges kix‡ii wewfbœ Ask wb‡q [Reproductive organs/body parts] ......11

Ab¨vb¨ (wbw`©ó K‡i D‡jøL Kiæb) [Other: specify] …............ 777

Rvwbbv [DK]…………………………………………………............. 99

- 1. gvwmK PjvKvjxb mg‡q hw` †g‡q‡`i kvwiwiK A¯^w¯Í/e¨v_v nq Zvn‡j G¸‡jv Kgv‡bvi Rb¨ Avcwb wK b~b¨Zg wZbUv c×wZ ej‡Z cvi‡eb? **(GKvwaK DËi MÖnb‡hvM¨; DËi c‡o †kvbv‡bv hv‡e bv)**[Can you list at least three methods for reducing pain if a girl experiences any physical discomfort during her period? **(Multiple answer allowed here; Don’t read the answer)**]…..........................................................................................……

e¨v_vi Jla w`‡q [Pain medication] ....... 1

e¨vqvg K‡i [Exercise] .......................... 2

Mig †mK/fvuc w`‡q [Hot fomentation] .... 3

VvÛv †mKw`‡q [Cold fomentation] ........ 4

Nywg‡q [Sleeping] ................................. 5

wekÖvgK‡i [Rest] ................................. 6

Ab¨vb¨ (wbw`©ó K‡i wjLyb)[Others (specify)]… 77

- 1. c~b©e¨envi‡hvM¨ m¨vwbUvix c¨vW (Kvco) ‡aŠZ Kiv Ges ïKv‡bvi mwVK Dcvq wK?**(DËi c‡o †kvbv‡bv hv‡e bv Ges GKvwaK DËiMÖnb‡hvM¨)** [What is the proper way to wash and dry reusable sanitary products (cloths)? **(Don’t read the answer and multiple answer allowed here)**]…...............................................................................................................…

cvwb w`‡q ‡aŠZ Kiv[Wash with water]................................................. 1

mvevb I cvwb w`‡q ‡aŠZ Kiv [Wash with water and soap]....................... 2

Kvco Ggbfv‡e ïKv‡Z w`‡Z n‡e †hb GwU m¤ú~b©fv‡e ïwK‡q hvq

[Cloths are dried in such a way that they can dry completely].......... 3

Ab¨vb¨ (wbw`©ó K‡i wjLyb)[Others (specify)]………………………….… 77

Rvwbbv [DK]………………………………………………...…........... 99

- 1. e¨eüZ m¨vwbUvix c¨vW (Kvco) †d‡j †`qvi mwVK Dcvq ‡KvbwU **(DËi c‡o †kvbv‡bv hv‡e Ges GKvwaK DËi MÖnb‡hvM¨)**? [What is the proper way to dispose of disposable sanitary products? **(multiple responses allowed here) (Response options should be read aloud)**….....................................................……

Wv÷web G †d‡j †`qv [Dispose in the waste bin].................................. 1

gywo‡q Wv÷web G †d‡j †`qv[Wrapped and placed in a dustbin]......... 2

j¨vwUª‡bi M‡Z© †d‡j †`qv[Tossed into the pit of the latrine]................... 3

Lv‡j †d‡j †`qv[Tossed in a canal]....................................................... 4

cywo‡q ‡djv [Incinerated]..................................................................... 5

evB‡i ‡Sv‡ci g‡a¨ ‡d‡j †`qv [Thrown outside in the bush].................... 6

AvBwmwWwWAvviwei m¨vwbUvix c¨vW wm‡÷g e¨envi Kiv [icddrb/DSK system].... 7

Ab¨vb¨ (wbw`©ó K‡i wjLyb)[Others (specify)]………………………….… 77

- 1. KZ Nb Nb gvwm‡Ki mgq e¨eüZ `ªe¨wU cwieZ©b Kiv DwPZ **(DËi c‡o †kvbv‡Z n‡e Ges GKvwaK DËi MÖnb‡hvM¨ bq)?** [How often should sanitary products be changed? **(Read response options aloud and the girl should select one answer)**]………………………………………

i³ ‡ei nIqvi cwigvb Abyhvqx hLbB cÖ‡qvRb nq, Z‡e AšÍZ cÖwZ Qq N›Uv cici [As needed, according to blood flow, but AT LEAST every six hours]………………………………………..1

cÖwZ N›Uvq, i³ ‡ei nIqvi cwigvb Abyhvqx [Every hour, regardless of blood flow]……………2

w`‡b GKevi[Once per day]……………………………………………………………….3

hLbB cÖ‡qvRb nq, Z‡e AšÍZ cÖwZ bq N›Uv cici [As needed, but AT LEAST every 9 hours]..4

Ab¨vb¨ (wbw`©ó K‡i wjLyb)[Others (specify)]………………………………………….… 77

- 1. wb¤œwjwLZ A½wUi Qwe †_‡K wewfbœ As‡ki bvg¸‡jv wK Avcwb wPwýZ Ki‡Z cvi‡eb? **(DËi`vZv‡K QwewU †`Lvb Ges †hUv †hUv ej‡Z cv‡i Zvi Dci ‡KvW Kiæb, hw` DËi`vZv GKwUI bv ej‡Z cv‡i Zvn‡j “RvwbbvÓ †KvW Kiæb )** [Could you please identify the name of the bodypart from the following picture?**(Please show the photo to respondent and tick whether she could mention following; If respondent can’t say any then tick on “99”**]

1. wW¤^vkq [Ovaries]
2. d¨v‡jvwcqvb wUDe (wW¤^K bvjx)[Fallopian tubes]
3. Rivq y[Uterus]
4. ‡hvwb [Vagina]

99. Rvwbbv [Don’t know]

**Image for students**

Avgv‡`i‡K mgq †`qvi Rb¨ Avcbv‡K ab¨ev` [***Thank you so much for your time]***
